# Interplay between proinflammatory cytokines, miRNA, and tissue lesions in *Anisakis*-infected Sprague-Dawley rats

**DOI:** 10.1371/journal.pntd.0007397

**Published:** 2019-05-15

**Authors:** Jerko Hrabar, Željka Trumbić, Ivana Bočina, Ivana Bušelić, Anamarija Vrbatović, Ivona Mladineo

**Affiliations:** 1 Laboratory of Aquaculture, Institute of Oceanography and Fisheries, Split, Croatia; 2 Department of Marine Studies, University of Split, Split, Croatia; 3 Faculty of Science, University of Split, Split, Croatia; Instituto de Ciências Biológicas, Universidade Federal de Minas Gerais, BRAZIL

## Abstract

**Background:**

Anisakiasis is an emerging public health problem, caused by *Anisakis* spp. nematode larvae. Anisakiasis presents as variable and unspecific gastrointestinal and/or allergic clinical symptoms, which accounts for the high rate of misdiagnosed cases.

**Methodology/Principal findings:**

The aim of this study was to characterize the early cellular (6–72 h p.i.) and molecular (6 h p.i.) immune response and general underlying regulatory mechanism in *Anisakis* infected rats. Each Sprague-Dawley rat was infected with 10 *Anisakis* spp. larvae by gastric intubation. Tissues with visible lesions were processed for: i) classic histopathology (HE), immunofluorescence (CD3, iNOS, S100A8/A9), and transmission electron microscopy (TEM); ii) target genes (*Il1b*, *Il6*, *Il18*, *Ccl3*, *Icam1*, *Mmp9*) and microRNA (Rat Immunopathology MIRN-104ZF plate, Quiagen) expression analysis; and iii) global DNA methylation. Histopathology revealed that *Anisakis* larval migration caused moderate to extensive hemorrhages in submucosal and epimysial/perimysial connective tissue. In stomach and muscle, moderate to abundant mixed inflammatory infiltrate was present, dominated by neutrophils and macrophages, while only mild infiltration was seen in intestine. Lesions were characterized by the presence of CD3^+^, iNOS^+^, and S100A8/A9^+^ cells. The greatest number of iNOS^+^ and S100A8/A9^+^ cells was seen in muscle. *Il6*, *Il1b*, and *Ccl3* showed particularly strong expression in stomach and visceral adipose tissues, but the order of expression differed between tissues. In total, three miRNAs were differentially expressed, two in stomach (*miRNA-451* and *miRNA-223*) and two in intestine (*miRNA-451* and *miRNA-672*). No changes in global DNA methylation were observed in infected tissues relative to controls.

**Conclusions/Significance:**

*Anisakis* infection induces strong immune responses in infected rats with marked induction of specific proinflammatory cytokines and miRNA expression. Deciphering the functional role of these cytokines and miRNAs will help in understanding the anisakiasis pathology and controversies surrounding *Anisakis* infection in humans.

## Author summary

Anisakiasis is a zoonotic disease (infection transmitted between animals and humans) contracted by consumption of raw or undercooked seafood contaminated with *Anisakis* spp. nematode larvae. Anisakiasis usually presents with variable and unspecific gastrointestinal and/or allergic symptoms, which accounts for the high rate of misdiagnosed cases. Due to changes in dietary habits, such as eating raw or undercooked seafood, anisakiasis is considered an emerging public health problem. Despite the increase in number of reported cases worldwide, mechanisms of immune response to this unspecific human pathogen are poorly known. We have shown that in experimentally infected rats, *Anisakis* larvae cause severe hemorrhages and necrotic changes of affected tissues in the early phase of infections. Neutrophils and macrophages were abundantly present in tissue lesions, while eosinophils, hallmark of helminth infections, were scarcely present. We have also demonstrated particularly strong expression of several inflammatory genes. Moreover, we give for the first-time insight into putative regulatory mechanism mediated via a distinct class of RNA molecules. Our study may provide new opportunities for better understanding of cellular and molecular response to *Anisakis* spp., aiming at development of more specific therapeutics and alleviation of pathologies associated with *Anisakis* spp. infection.

## Introduction

Foodborne parasitic diseases have received increasing attention in recent decades. Some are considered emerging public health issues, due to changes in various factors influencing the propagation of parasites that cause them [1]. This is especially valid for several helminthiases, which have seen a rise in reported cases outside the natural range of their causative agents, mostly due to changes in dietary habits, such as eating raw or undercooked food, and increased demand and availability of “exotic” food [1,2]. The Foodborne Disease Burden Epidemiology Reference Group (FERG) published a list of parasites that could be transmitted to humans by food. The list includes several helminth taxa considered to present substantial disease burden, such as *Fasciola* spp., *Trichinella* spp., *Echinococcus* spp., *Clonorchis* spp., *Opistorchis* spp., *Taenia solium*, *Anisakis simplex*, and *Ascaris lumbricoides* [3]. Although *A*. *simplex* was subsequently removed from the priority list as being an uncommon foodborne parasite [4], the European Food Safety Authority (EFSA) has listed anisakid nematodes as parasites of high public health importance [5]. In addition, a recent study ranked Anisakidae within the top 10 and top 5 foodborne parasites important in Northern and South-Western Europe regions, respectively [6]. This is also evidenced from the recently published analysis of a 10-year hospital discharge records that reported 370 confirmed cases of anisakiasis in Italy, with an annual incidence of 1.5 to 15.5 per 100000 hospitalizations [7]. Conversely, only 37 cases of anisakiasis were estimated over a 5-year period in France based on national survey among hospital Parasitology laboratories in France[8]. However, a recent risk assessment analysis indicated a substantial underestimation of *Anisakis* infection in European countries, predicting 7700–8320 annual cases for Spain only [9].

Members of the genus *Anisakis*, (with currently nine valid species) are parasitic nematodes with an indirect life cycle that utilize marine mammals, primarily cetaceans, as definitive hosts, planktonic crustaceans as first intermediate hosts, and fish and cephalopods as paratenic hosts [10]. In definitive hosts, these parasites induce chronic changes in the gastrointestinal mucosa ranging from superficial erosions to deep ulcerations. Histopathologically, lesions are characterized by lymphoplasmacytic gastritis, granulomatous or eosinophilic inflammation with the presence of multinucleated giant cells, hemosiderosis, and fibrosis [11–14]. Following ingestion of raw or undercooked seafood infected with live *Anisakis* third-stage larvae (L3), humans can become accidental hosts; thus contracting a disease termed anisakiasis [2,15]. In humans, *Anisakis* larvae cannot reach adult stage, and although infection can be asymptomatic, it is often followed by an acute clinical state. A recent classification lists four types of disease: gastric, intestinal, ectopic (extragastrointestinal), and allergic (gastro-allergic) anisakiasis [16–18]. Gastric anisakiasis is characterized by redness and swelling at the site of larval migration, hemorrhages, erosive gastritis, and mucosal ulceration with mixed inflammatory infiltrate dominated by eosinophilic granulocytes [17]. Intestinal anisakiasis is characterized by mucosal edema and luminal stenosis; rarely intestinal obstruction and intussusception occur [17,19]. Chronic infection can result in formation of abscesses, ulcers, and eosinophilic granulomas forming around dying larva [17,20]. Such histopathological changes are caused by mechanical tissue damage during larval migration as well as secreted serine proteases and metalloproteases that degrade collagen and glycoproteins to facilitate larval migration [21,22]. In addition, such changes may also arise from the cytotoxic effect of eosinophilic granulocytes that accumulate in high numbers around the lesion [16].

Helminth infections in both humans and animals are characterized by induction of the T helper 2 (T_H_2) immune response, with the exception of species of the genus *Trichuris*, which elicit mixed T_H_1/T_H_2 response [23]. Although different types of parasite induce the T_H_2 response, effects of such a response can differ greatly either resulting in worm expulsion or downregulation of T_H_1 and T_H_17 thus preventing associated immunopathology [24]. The hallmark of the T_H_2 response is the production of type 2 cytokines such as interleukin-4 (IL-4), IL-5, IL-9, and IL-13, goblet cell (GC) hyperplasia and subsequent mucus production, mastocytosis, expansion of eosinophils and basophils as well as alternatively activated macrophages (AAM), which contribute to fibrosis and tissue repair [25–27]. Although CD4^+^ cells are central players in the T_H_2 response, basophils and type 2 innate lymphoid cells are considered important primary sources of IL-4 and IL-13, respectively. These cytokines act in autocrine fashion to stimulate T_H_2-cells to reinforce their own production [25,28]. The majority of parasites establish chronic infections by downregulating host immune response, which is also beneficial for the host, as it diminishes related immunopathology [23]. In contrast, zoonotic infections where humans are accidental hosts, such as with anisakiasis, can result in more pronounced immune response and associated pathology [29].

Immune responses to pathogens can be regulated by several mechanisms, either post-transcriptionally via non-coding RNAs or pre-transcriptionally by modifying DNA (acetylation, methylation). MicroRNAs (miRNAs) are a class of small non-coding RNAs, ~22 nt long, involved in post-transcriptional regulation of gene expression. [30,31]. A single miRNA can regulate more than one transcript and numerous miRNAs can regulate a single transcript. The involvement of specific miRNAs in immune responses to intracellular [32] and extracellular parasites [33] has been documented. DNA methylation represses gene expression by adding methyl groups to CpG dinucleotides in promoter regions of genes; thus, preventing binding of transcription factors [34].

To date, research on murine models of anisakiasis has focused mainly on chronic infection and allergic reactions, leaving a gap in our knowledge of the early immune response to *Anisakis* infection. Although reports of histopathological changes induced by *Anisakis* spp. larvae exist both from experimental anisakiasis [35] as well as human clinical cases (e.g. [36]), such studies employed only conventional histological techniques (i.e. hematoxylin-eosin staining) lacking immunophenotyping of cellular infiltrate or the ultrastructural details of observed histopathological changes.

Therefore, the aim of this study was to characterize the early immune response to *Anisakis* infection in rats both at cellular and molecular levels by i) assessing histopathological changes using three different approaches (i.e., classic histopathological analysis, immunofluorescence and transmission electron microscopy); ii) measuring expression of a set of common inflammatory markers and their putative regulatory miRNAs in tissues where larval migration was observed and, iii) screening for changes in global DNA methylation in infected tissues.

## Materials and methods

### Ethics statement

*In vivo* experiments on rats were performed at the University of Split Animal Facility (permit number HR-POK-19). The study was approved by Veterinary and Food Safety Authority, Ministry of Agriculture of the Republic of Croatia (permit number EP 18-2/2016) and Ethics Committee of the University of Split, School of Medicine (permit number 003-08/18-03/0001). All animal care and use protocols applied in this study adhere to Section three—Protection of animals used for scientific purposes of the Animal Protection Act of the Republic of Croatia (NN 102/17), which implements guidelines of the Directive 2010/63/EU of the European Parliament and of the Council on the protection of animals used for scientific purposes (SL L 276, 20.10.2010).

### *Anisakis* experimental infection of rats

Animals were raised and housed in plastic cages with sawdust bedding in a controlled environment (temperature 22 ± 1°C, 12 h dark/light cycle, food and water ad libitum) and were deprived of food 12 h prior to experimental infection.

Experimental *Anisakis* infection was performed on 10 male Sprague-Dawley rats (average weight 360.2 g ± SD 86.67 g) and tissue sampling for target gene and microRNA expression and methylated DNA quantification was set to 6 h post-infection (p.i.) based on the results from preliminary experiment performed on 15 female Sprague-Dawley rats (average weight 197 g ± SD 13.6 g). The preliminary experiment has been previously described in detail in [37]. The samples for histopathological analysis, immunofluorescence, and transmission electron microscopy were collected during the preliminary experiment (6, 10, 24, 48, 72 h p.i.) and used exclusively in this study. Prior to experimental infection, animals were anesthetized with a mixture of anesthetic and analgesic by intraperitoneal injection, 50–100 mg/kg Ketaminol (Richter Pharma AG, Wels, Austria), and 5–10 mg/kg Xylapan (Vetoquinol UK Ltd, Buckingham, UK). When no toe pinch reflex was detected, each animal was orally intubated with 10 live *Anisakis* larvae, collected from blue whiting *Micromesistius poutassou*, according to a previously described protocol [35]. Animals were euthanized by an overdose of anesthetic (> 150 mg/kg) followed by decapitation to confirm death. Each animal was dissected and inspected for lesions caused by migrating *Anisakis* larvae. Tissues with visible lesions with or without *larva migrans* were sampled and stored appropriately for downstream analyses (S1 Table). Additionally, adjoining unaffected tissue was sampled and served as an internal control in order to reduce interindividual variability. No external control (uninfected, “healthy” rats) was used in this study, considering that principal component analysis (PCA) results from our previous study showed higher variability between such specimens within the same experimental groups [37].

### Histopathology of *Anisakis* lesions

For histopathological analysis, tissue samples of *Anisakis* lesions from preliminary experiments were fixed in 4% paraformaldehyde on ice, processed using standard techniques, paraffin-embedded and cut to 5 μm thickness. Tissue sections were HE stained, mounted in Canada balsam (Sigma, St. Louis, MO, US), coverslipped and evaluated for histopathological changes using an Olympus CX40 microscope (Olympus Corp., Shinjuku, Tokyo, Japan). Photos were captured with an Olympus Camedia camera (Olympus Corp.) and assembled with Photoshop CS5 software (Adobe Systems, San Jose, CA, USA).

### Immunofluorescence

Cluster of differentiation 3 (CD3), inducible nitric oxide synthase (iNOS) and S100A8/A9 (calgranulin A and B, calprotectin as heterodimer; migration inhibitory factor-related protein 8 and 14) were chosen as markers for immunofluorescent evaluation of *Anisakis* lesions. CD3 is a pan T-cell marker, which associates with the T-cell receptor (TCR) to activate different subsets of T cells [38]. iNOS is one of three nitric oxide synthases (the other two being neuronal [nNOS] and endothelial [eNOS]) that is not constitutively expressed but strongly upregulated in many cell types in response to various pathogens [39]. S100A8 and A9 are alarmins or DAMPs (damage-associated molecular pattern proteins) that are secreted from damaged cells or activated granulocytes and monocytes, and play a pivotal role in mediating the inflammatory response [40]. Immunofluorescence staining was performed on 4% paraformaldehyde fixed, paraffin-embedded tissue samples from the preliminary experiment. Tissue sections (5 μm) were placed on FLEX IHC microscope slides (Dako, Glostrup, Denmark), deparaffinized and rehydrated. Antigen retrieval was performed by incubating sections at 95°C in Dako Antigen Retrieval Solution, pH = 6.0 (Dako) for 20 min. Following cooling to room temperature, sections were incubated in 1% BSA in PBS (Sigma, St. Louis, MO, US) at room temperature to prevent non-specific binding. Due to high erythrocyte autofluorescence in the green channel, Image-iT FX signal enhancer (Invitrogen, Carlsbad, CA, US) was applied to slides stained with an Alexa Fluor488-conjugated secondary antibody for 30 min at room temperature and washed with Dako Wash Buffer (TBS-Tween 20) (Dako) prior to BSA blocking. Sections were then incubated with rabbit anti-CD3 epsilon antibody (1:200, ab49943, Abcam, Cambridge, UK), rabbit anti-iNOS antibody (1:100, ab15323, Abcam) and mouse anti-MRP8+MRP14 [B314.1 (MAC 387)] (1:100, ab130234, Abcam) for 1 h at room temperature. Subsequently, sections were washed in Dako Wash Buffer and incubated with donkey anti-rabbit IgG H&L (Alexa Fluor594) (2 μg/ml, ab150068, Abcam) and goat anti-mouse IgG H&L (Alexa Fluor488) (1:200, ab150117, Abcam), respectively, for 1 h at room temperature. Finally, sections were washed with TBS, counterstained with DAPI, and coverslip mounted using Shandon Immu-Mount (Thermo Scientific, Waltham, MA, US). As a negative control, primary antibodies were omitted (Supporting information). Sections were inspected under a Zeiss Axio Imager M1 fluorescence microscope equipped with AxioCam Mrm Rev 3 (Carl Zeiss AG, Oberkochen, Germany). Images were captured with AxioVision Rel. 4.7 software (Carl Zeiss AG) and assembled with Photoshop CS5 software (Adobe Systems).

### Transmission electron microscopy

For TEM, small pieces of *Anisakis*-induced lesions from the preliminary experiment were fixed in 4% paraformaldehyde on ice and post-fixed in 1% aqueous osmium tetroxide. In-block staining with 2% aqueous uranyl acetate was performed overnight, followed by dehydration in an ascending series of acetone and embedding in Durcupan resin (Honeywell-Fluka, Morris Plains, NJ, US). Semi-thin sections were cut to 500 μm, stained with 1% toluidine blue and inspected under a light microscope for orientation. Ultrathin sections were cut at 0.07 μm, stained with uranyl acetate and lead citrate [41], and inspected under a Jeol JEM-1400 TEM operating at 120 kV.

### RNA extraction, target gene and miRNA expression

Total RNA was extracted using TriReagent (Ambion Inc., Invitrogen, Carlsbad, CA, USA) following the manufacturer’s protocol and dissolved in 20–40 μL of Mili-Q water (Merck Millipore, Billerica, MA, US). The quantity and quality of extracted RNA were checked by spectrophotometry and 1% agarose gel electrophoresis, respectively. Prior to cDNA synthesis, RNA samples were treated with *DNA-*free *DNA Removal Kit* (Invitrogen, Carlsbad, CA, USA) to avoid amplification of genomic DNA in the downstream analysis.

For target gene expression, cDNA was synthesized from 1 μg of total RNA using a *High-Capacity cDNA Reverse Transcription Kit* (Applied Biosystems, Waltham, MA, US) following the manufacturer’s protocol. Target genes were selected based on recently published transcriptomic data [37] and evidence for their regulation by miRNAs. The target genes are interleukin 1 beta (*Il1b*), *Il6*, *Il18*, chemokine (C-C motif) ligand 3 (*Ccl3*), intracellular adhesion molecule 1 (*Icam1*), and matrix metallopeptidase 9 (*Mmp9*). Expression was quantified by real-time PCR using LightCycler 480 SYBR Green I Master (Roche Diagnostics, Manheim, Germany) with cycling conditions as previously described [42] and annealing set to 60°C. Vacuolar protein sorting-associated protein 29 (*Vps29*) and glucose-6-phosphate isomerase (*Gpi*) were selected as reference genes based on their stability in recent transcriptome [37] and BestKeeper [43] analyses. Prior to real-time PCR, the template cDNA was diluted 1:20 with MilliQ water and each sample was run in duplicate. Primer3 (v. 4.1.0.) web interface [44] was used to create specific primers (Table 1).

**Table 1:** Oligonucleotide primers used for gene expression analysis.

	Primer name	Nucleotide sequence (5’ → 3’)
Interleukin-1β (*Il-1β*)	Rno_Il1B_F Rno_Il1B_R	TCAAGCAGAGCACAGACCTG ACTGCCCATTCTCGACAAGG
Interleukin-6 (*Il-6*)	Rno_Il6_F Rno_Il6_R	GCAAGAGACTTCCAGCCAGT TCTGACAGTGCATCATCGCT
Interleukin-18 (*Il-18*)	Rno_Il18_F Rno_Il18_R	AGGACTGGCTGTGACCCTAT TCCTGGCACACGTTTCTGAA
Matrix metallopeptidase 9 (*Mmp9*)	Rno_Mmp9_F Rno_Mmp9_R	CCCGAGACCTGAAAACCTCC GGCCTTTAGTGTCTCGCTGT
Intracellular adhesion molecule-1 (*Icam-1*)	Rno_Icam1_F Rno_Icam1_R	CGGTGCTCAGGTATCCATCC CTGTCTTCCCCAATGTCGCT
Chemokine (C-C motif) ligand 3 (*Ccl3*)	Rno_Ccl3_F Rno_Ccl3_R	CACCCTCTGTTACCTGCTCA ATCTGCCGGTTTCTCTTGGT
Vacuolar protein sorting-associated protein 29 (*Vps29*)	Rno_Vps29_F Rno_Vps29_R	TGGTGACTGAACGGAATCCC GGACATCACCAGCCAGAGTC
Glucose-6-phosphate isomerase (*Gpi*)	Rno_Gpi_F Rno_Gpi_R	GACGTGATGCCAGAGGTCAA GTTTTGGCAATGTGGGTCCC

For miRNA expression, cDNA was synthesized using *miScript II RT Kit* (Qiagen, Hilden, Germany) following the manufacturer’s protocol. miRNA expression was evaluated on *miRNA PCR array Rat Immunopathology (MIRN-104ZF)* plates with *QuantiTect SYBR Green Master Mix* (Qiagen, Hilden, Germany). All real-time PCR experiments were run on a LightCycler 480 II System (Roche Diagnostics, Rotkreuz, Switzerland).

### DNA extraction and global DNA methylation quantification

For DNA extraction, tissue samples were stored in 96% EtOH at 4°C until analysis. Total genomic DNA was extracted using a DNeasy Blood and Tissue Kit (Qiagen, Hilden, Germany). The quality and quantity of extracted DNA were checked as mentioned previously.

Global DNA methylation was quantified using a *Methylated DNA Quantification Kit (Colorimetric)* (ab117128, Abcam, Cambridge, UK), following the manufacturer’s protocol. The plate was read on a Microplate Photometer MPP-96 (Biosan, Riga, Latvia).

### Statistical analysis

Log2 transformation and differential expression analysis of miRNA and target genes was performed with the *limma* package [45] for R (ver. 3.4.2) [46]. Target genes expression was normalized on individual basis against the geometric mean of two housekeeping genes (*Vps29* and *Gpi*). Sample RN10_3K (intestine, uninfected) was excluded from further expression analysis since explorative analysis showed this sample to be an outlier. Expression analysis for both miRNA and target genes was run separately for each tissue using a paired design, which considers that uninfected (control) tissue was sampled from the same animal as infected tissue. Fold changes were calculated relative to the uninfected tissue as a group. Fold-change greater than 2 (log2FC ≥ 1) was considered biologically significant, while statistical significance was set to adjusted *p*-value < 0.05. Biological significance is regarded as a change high enough to result in certain outcome, i.e. change in gene expression that could result in enough protein secretion to manifest a certain effect. Target genes and miRNA expression analysis results were visualized with ggplot2 [47] and gplots [48] for R, respectively.

To test for differences in global DNA methylation between infected and uninfected tissue of rat stomach and intestine regarding alterations in global DNA methylation, Wilcoxon signed-rank test was performed in R. This non-parametric statistical hypothesis test is suitable for comparison of mean ranks between paired samples, as is the case in our sampling design. It does not require the assumption of a Gaussian distribution.

## Results

Previous study published by same authors demonstrated a short *Anisakis* spp. infection duration in rats, with majority of introduced larvae (~70%) leaving the host within the first 24 h post-infection (p.i.) either by passage through the gastrointestinal tract with no visible tissue damage or by migration through the gastrointestinal wall [37]. The majority of migrating larvae penetrated the gastric wall, causing moderate to severe hemorrhages, with the remainder penetrating small intestine, cecum or colon. In three animals (two from the preliminary experiment and one from the main experiment) *Anisakis* spp. larvae were found penetrating abdominal wall musculature, causing mild hemorrhages visible upon gross pathological examination. The larvae that were not expelled with feces were found dwelling in the abdominal cavity, either migrating through the abdominal adipose tissues or in the process of spiralization on the surface of internal organs (liver, spleen).

### Histopathology of *Anisakis* lesions

**Fig 1:**
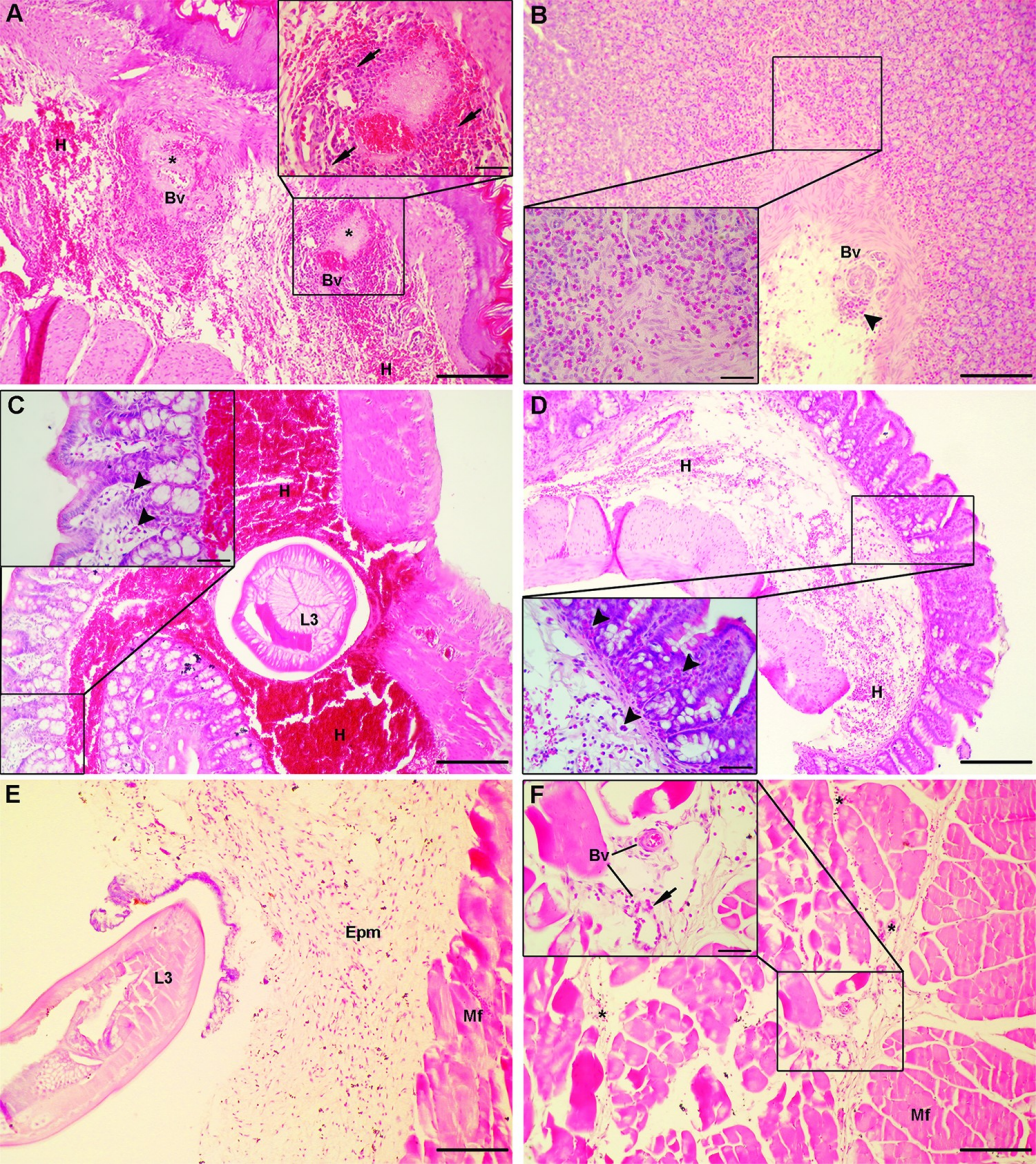
Histopathological findings in rats infected with *Anisakis* spp. L3. (A) Severe submucosal hemorrhage (H) in stomach at the stomach-esophageal junction, adjacent to the site of larval penetration with two necrotic foci (asterisks) around large blood vessels (Bv) (HE, 100x); (a) inset: high-power magnification of a necrotic focus showing vascular and perivascular necrosis, surrounded by abundant neutrophil and macrophage infiltrate (arrows) (400x); (B) Stomach of a rat euthanized 24 h p.i. with abundant eosinophil infiltrate in *lamina propria* and around blood vessels (Bv) (arrowhead) indicating recent extravasation (HE, 100x); (b) inset: detail of abundant eosinophil infiltrate (400x); (C) Severe submucosal hemorrhage (H) in intestine with a cross-section of *Anisakis* spp. larva (L3) (HE, 100x); (c) inset: detail of mucosa with rare eosinophils in *lamina propria* (arrowheads) (HE, 400x); (D) Extensive submucosal hemorrhage (H) in caecum (HE, 100x); (d) inset: detail of mucosa with abundant eosinophil infiltration (arrowheads) in *lamina propria* (HE, 400x); (E) Migration of *Anisakis* spp. larvae (L3) through epimysial connective tissue (Epm) causing moderate to extensive hemorrhage accompanied by mixed inflammatory infiltrate, composed mostly of neutrophils and macrophages (Mf = muscle fibers) (HE, 100x); (F) Muscle tissue adjacent to site of larval migration showing mild perimysial hemorrhage (asterisks) and focal necrosis of muscle fibers (Mf) (HE, 100x); (f) inset: detail of perimysial connective tissue with two blood vessels (Bv) surrounded by moderate neutrophil infiltrate (arrow) (400x). Scale bar (A, B, C, D, E, F) = 200 μm, scale bar (inset) = 50 μm.

Larval penetration caused moderate to severe submucosal hemorrhage in stomach (Fig 1A), with focal to confluent fibrinoid necrosis in rare cases (Fig 1A inset). Moderate to abundant mixed inflammatory infiltrate composed of neutrophils, macrophages, lymphocytes, and eosinophilic granulocytes was seen. In animals euthanized at the first two time points (6 h and 10 h p.i.), neutrophils were the predominant cell population in inflammatory infiltrate, with scant eosinophilic granulocytes interspersed between zymogenic cells and *muscularis mucosae*. Lesions from animals euthanized at later time points (24 h, 48 h and 72 h p.i.) were characterized by more prominent mucosal and submucosal eosinophilic granulocyte infiltration (Fig 1B and 1B inset) with occasional neutrophils. In the intestine, *Anisakis* spp. larvae were found penetrating submucosal tissue and causing severe hemorrhage with perivascular fibrinoid necrosis (Fig 1C). Scant inflammatory infiltrate composed of neutrophils, macrophages, and lymphocytes was seen in submucosal tissue, while the mucosal layer was infiltrated with scant eosinophilic granulocytes (Fig 1C inset). In contrast to the intestine, moderate to extensive hemorrhage was seen in the caecum (Fig 1D) with more pronounced inflammatory infiltrate composed of neutrophils, macrophages, and lymphocytes and eosinophilic granulocytes infiltrating both mucosal and submucosal layers (Fig 1D inset). Larval migration through muscle tissue resulted in moderate hemorrhage in epimysial and perimysial connective tissue (Fig 1E) accompanied by mixed inflammatory infiltrate dominated by neutrophils and macrophages (Fig 1E inset), occasional eosinophilic granulocytes and scant lymphocytes. Muscle fibers showed signs of coagulative necrosis. Representative micrographs of uninfected control tissue are presented in S1 Fig.

### Immunofluorescence

**Fig 2:**
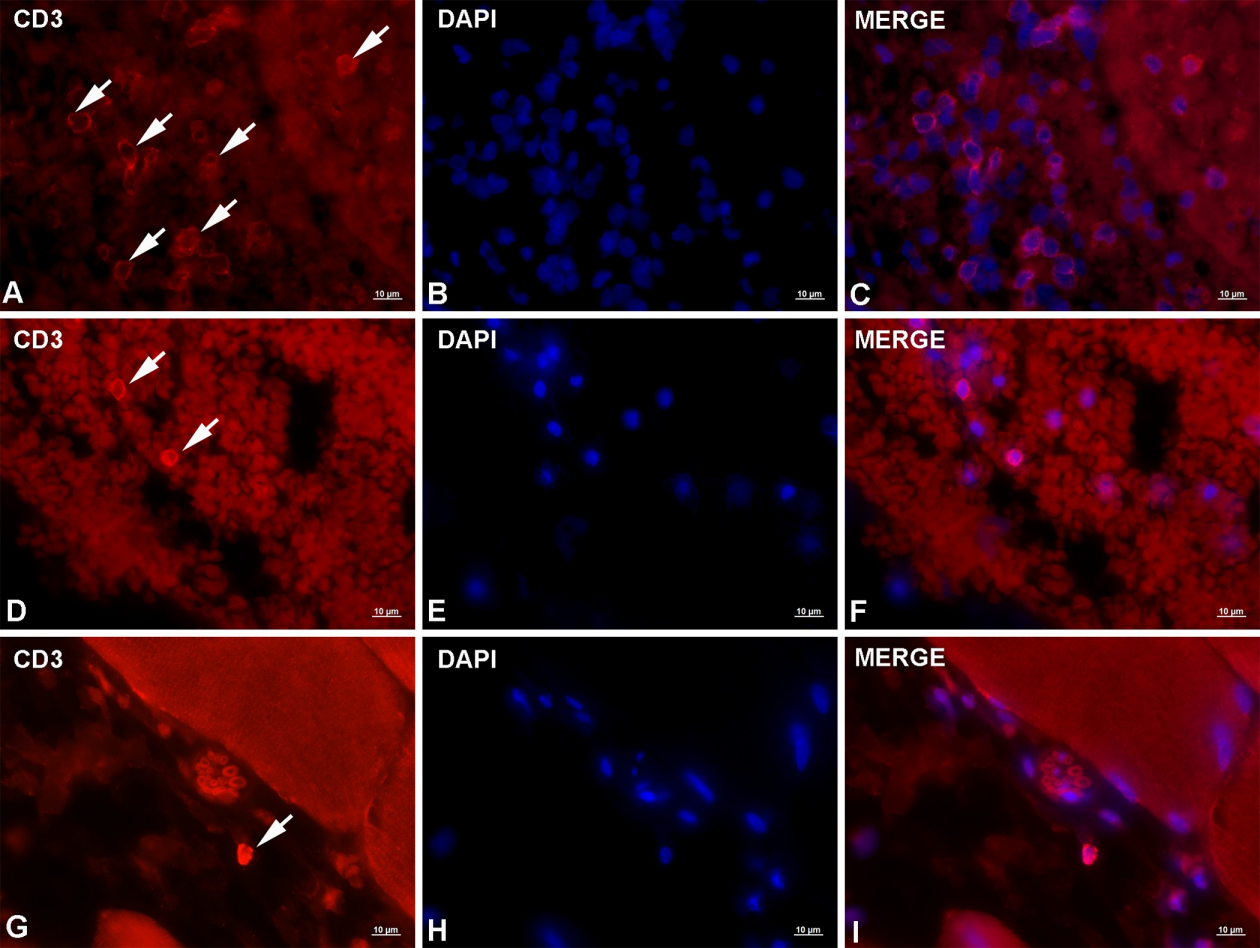
Representative micrographs of CD3 immunolabeling in rats experimentally infected with *Anisakis* spp. third-stage larvae. (A) Moderate CD3 expression on a large number of T lymphocytes (arrows) in stomach adjacent to blood vessel; (B) DAPI-stained cell nuclei; (C) Merge of (A) + (B); (D) Two lymphocytes in the intestinal submucosa showing strong CD3 expression (arrows); (E) DAPI-stained cell nuclei; (F) Merge of (D) + (E); (G) Single T lymphocyte with very strong CD3 expression in perimysial connective tissue (arrow); (H) DAPI-stained nuclei; (I) Merge of (G) + (H). Immunofluorescence, scale bar = 10 μm.

Immunofluorescent labeling of lesions caused by migrating *Anisakis* larvae showed positive staining for all three antibodies applied (i.e. anti-CD3, anti-iNOS, and anti-MRP8+MRP14 (anti-S100A8/A9)). Anti-CD3 staining produced moderate to strong signal with thin ring-like cytoplasmic staining corresponding to scarce cytoplasm of lymphocytes. CD3^+^ cells were most abundant in the stomach, usually seen in groups within or adjacent to larger blood vessels (Fig 2A, 2B, and 2C), indicating recent and ongoing extravasation. Occasionally, single CD3^+^ cells were observed deeper in the submucosa within large hemorrhages or very rarely in the *lamina propria*. In the intestine, only a few cells moderately expressing CD3 were observed in the submucosa with similar localization as in the stomach (Fig 2D, 2E, and 2F) (i.e. within or adjacent to blood vessels). The smallest number of CD3^+^ cells, but with strongest expression, was found in muscle tissue. Single cells were occasionally seen within blood vessels or infiltrating perimysial connective tissue (Fig 2G, 2H, and 2I). Uninfected control tissue and negative stain control tissue are presented in S2 Fig and S3 Fig, respectively.

**Fig 3:**
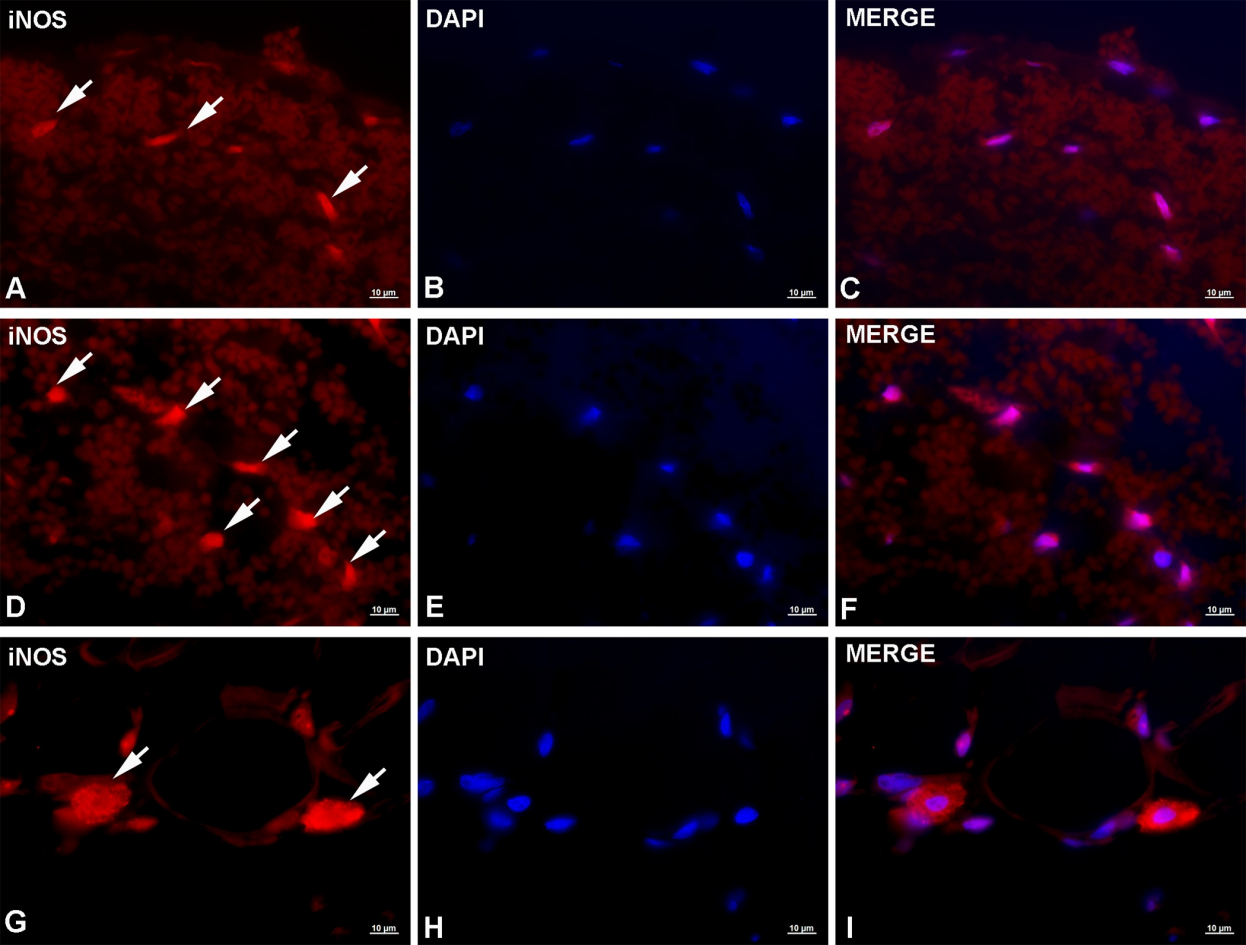
Representative micrographs of iNOS immunolabeling in rats experimentally infected with *Anisakis* spp. third-stage larvae. (A) Moderate to strong granular cytoplasmic expression of iNOS in a low number of cells in stomach (arrows); (B) DAPI-stained cell nuclei; (C) Merge of (A) + (B); (D) Strong iNOS expression in intestinal cells adjacent to migrating *Anisakis* larvae (arrows); (E) DAPI-stained cell nuclei; (F) Merge of (D) + (E); (G) Very strong iNOS expression in perimysial connective tissue in cells most likely belonging to the mononuclear phagocyte system, adjacent to blood vessel (arrows); (H) DAPI-stained nuclei; (I) Merge of (G) + (H). Immunofluorescence, scale bar = 10 μm.

Anti-iNOS immunolabeling produced moderate to intense signal, showing either granular or diffuse cytoplasmic staining. In stomach, individual or small groups of iNOS^+^ cells could be seen throughout extensive submucosal hemorrhages (Fig 3A, 3B, and 3C). Most of these cells were characterized by moderate granular cytoplasmic and perinuclear staining, with occasional cells yielding stronger signals. Based on cellular and nuclear morphology the most abundant iNOS^+^ cells appeared to be neutrophils and fibrocytes, however, no staining with specific markers has been performed to confirm cell types. In intestine, similarly to the stomach, iNOS^+^ cells were observed throughout the submucosal hemorrhage but tended to group around the migrating *Anisakis* larva. Again, various cell types were iNOS^+^ but yielded a stronger signal than their counterparts in stomach (Fig 3D, 3E, and 3F). The highest number of iNOS^+^ cells, showing the strongest expression, was seen in muscles. Most of the cells showed strong granular or diffuse cytoplasmic staining (Fig 3G, 3H, and 3I). As in the stomach and intestine, the most abundant iNOS^+^ cells appeared to be neutrophils and fibrocytes. A low number of cells, most likely mononuclear phagocytes, showing very strong, diffuse cytoplasmic staining could be seen randomly dispersed in the perimysium or adjacent to blood vessels. Uninfected control tissue and negative stain control tissue are presented in S4 Fig and S5 Fig, respectively.

**Fig 4:**
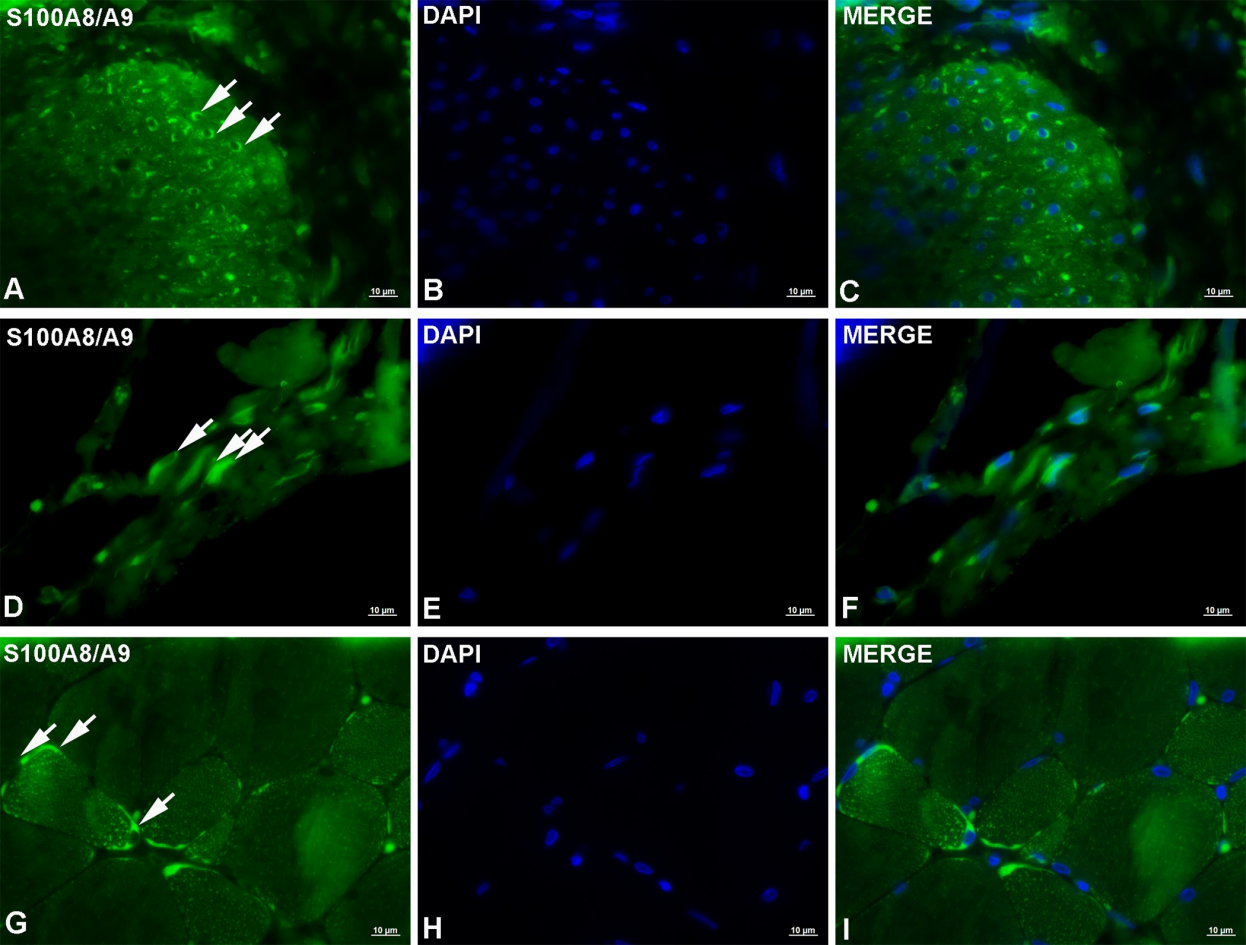
Representative micrographs of S100A8/A9 (MRP8+MRP14) immunolabeling in rats experimentally infected with *Anisakis* spp. third-stage larvae. (A) Strong diffuse cytoplasmic labeling of S100A8/A9 in a low number of cells in the inner layer of the *muscularis externa* of rat stomach (arrows); (B) DAPI-stained cell nuclei; (C) Merge of (A) + (B); (D) Moderate diffuse cytoplasmic expression of S100A8/A9 in endothelial cells in intestinal submucosa; (E) DAPI-stained cell nuclei; (F) Merge of (D) + (E); (G) Very strong sarcoplasmic expression of S100A8/A9 in abdominal muscle myocytes (arrows); (H) DAPI-stained nuclei; (I) Merge of (G) + (H). Immunofluorescence, scale bar = 10 μm.

Anti-MRP8+MRP14 (anti-S100A8/A9) staining yielded moderate to very strong granular or diffuse cytoplasmic signals. In the stomach of animals euthanized at earlier time points, a fairly low number of cells with moderate to strong expression was seen in *muscularis externa* (Fig 4A, 4B, and 4C) together with, most likely, rare fibrocytes in the submucosa and eosinophils in the *lamina propria*, respectively; however no staining with specific markers has been performed to confirm cell types. In animals euthanized at later time points, groups of eosinophils with high expression could be seen in the *lamina propria*. In the intestine, a low number of MRP8^+^MRP14^+^ cells were seen, mostly fibrocytes and endothelial cells (Fig 4D, 4E, and 4F). The highest number of MRP8^+^MRP14^+^ cells with strongest expression was seen in muscle. Numerous immunoreactive cells, including most likely fibrocytes, eosinophils, and neutrophils, yielding strong (rarely very strong) signals, could be seen in perimysium and epimysium. However, considerably stronger expression, occasionally exceptionally strong, was seen in myocytes, which were also the most abundant MRP8^+^MRP14^+^ cells (Fig 4G, 4H, and 4I). In longitudinal muscle fiber section, strong granular sarcoplasmic staining could be seen, while in cross-sections, cells yielded strong diffuse sarcoplasmic signals. Uninfected control tissue and negative stain control tissue are presented in S6 Fig and S7 Fig, respectively.

### TEM

**Fig 5:**
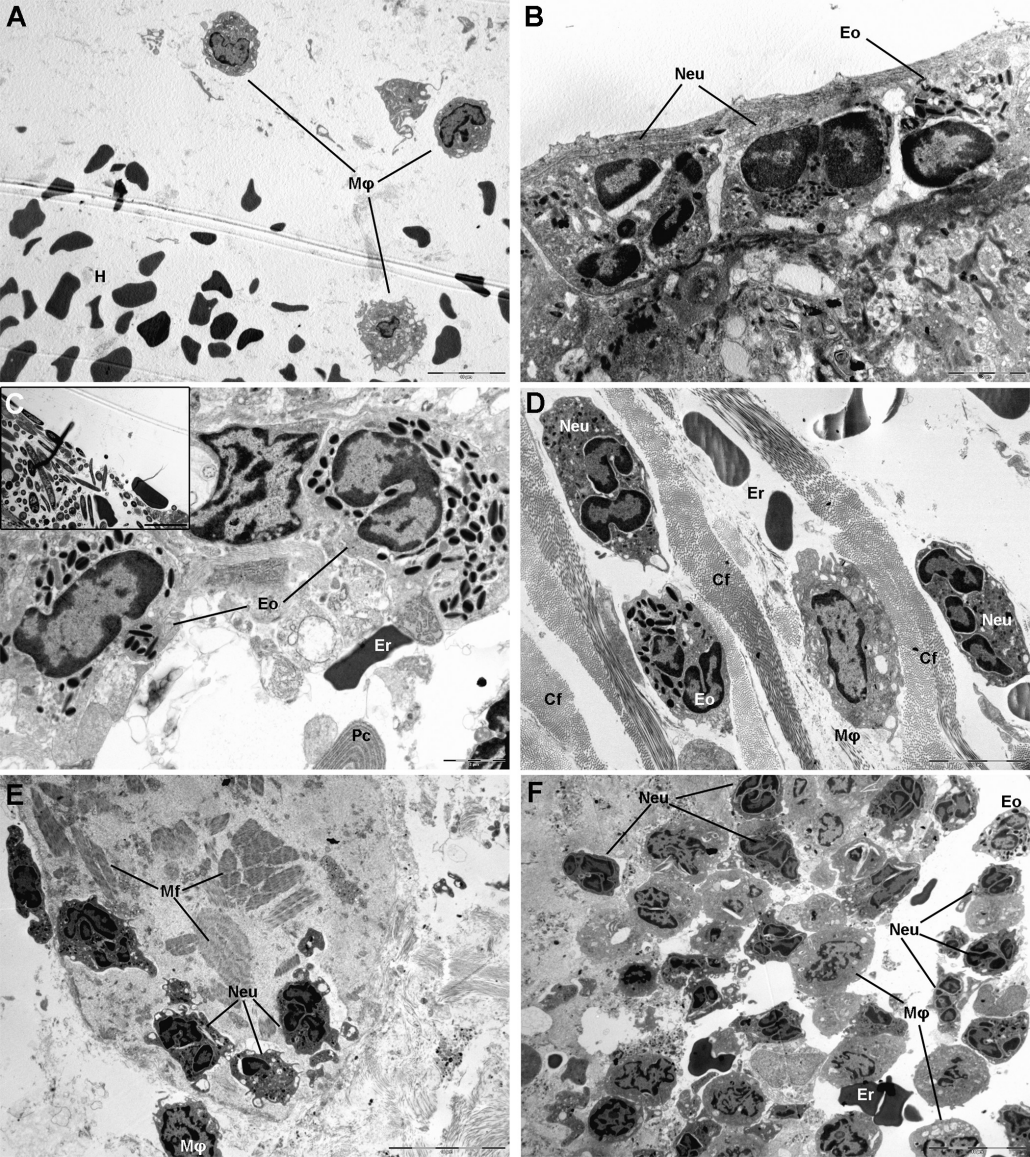
Transmission electron micrographs of lesions in rats experimentally infected with *Anisakis* spp. third-stage larvae. (A) Hemorrhage (H) in *lamina propria* of stomach with three macrophages (Mφ); (B) Rat stomach adjacent to site of larval migration through stomach wall with neutrophil (Neu) and eosinophil (Eo) infiltration; (C) Rat intestine adjacent to site of larval migration through intestinal wall with a single erythrocyte (Er) indicative of hemorrhage and eosinophil (Eo) and macrophage (Mφ) infiltration; bottom of picture shows elaborate endoplasmic reticulum indicative of a plasma cell (Pc). Inset: numerous elongated fungus-like organisms with electron lucent intracytoplasmic granules; (D) Mixed inflammatory infiltrate of rat caecum composed of neutrophils (Neu), eosinophils (Eo) and macrophages (Mφ) interspersed between collagen fibers (Cf) and mixed with non-nucleated erythrocytes (Er) indicating hemorrhage; (E) Necrotic myocytes of abdominal muscle with fragmented muscle fibers (Mf); few neutrophils with outspread pseudopodia migrating over muscle fibers (Mf) and a single macrophage (Mφ) are visible; (F) Mixed inflammatory infiltrate of rat muscle dominated by neutrophils (Neu), and few eosinophils (Eo), macrophages (Mφ), and erythrocytes (Er), indicating hemorrhage. Osmium tetroxide/lead citrate, scale bar: (A, E, F) = 10 μm, scale bar (B, C) = 2 μm, scale bar (D, C inset) = 5 μm.

TEM analysis of *Anisakis*-induced lesions revealed ultrastructural details of inflammatory infiltrate and tissues changes. As seen in paraffin sections, gastric lesions were characterized by hemorrhage and abundant neutrophil and macrophage infiltration (Fig 5A and 5B). The characteristic “coffee bean” structure of cytoplasmic granules evidenced the presence of eosinophils in inflammatory infiltrate and a lack of mast cells (Fig 5B). Macrophages with engulfed erythrocytes could occasionally be seen. In the intestine, neutrophils and eosinophils were seen mixed with erythrocytes and interspersed between bundles of collagen fibers. A single plasma cell, with elaborate endoplasmic reticulum, was seen associated with several eosinophils (Fig 5C). However, an unexpected finding of numerous fungus-like organisms (Fig 5C inset) with intraluminal and submucosal localization was noticed, some of which were endocytosed by eosinophils. The organisms were elongated and round in cross-section, several micrometers long (up to 9 μm) and with electron light intracytoplasmic granules. Similarly, neutrophil, macrophage, and eosinophil infiltration (lacking mast cells) was observed in caecum (Fig 5D). In muscle, several areas of structurally fragmented and necrotic fibers were observed (Fig 5E). These areas were heavily infiltrated with a mixture of neutrophils, macrophages, and eosinophils (Fig 5E), some with expelled granules.

### Target gene expression

**Fig 6:**
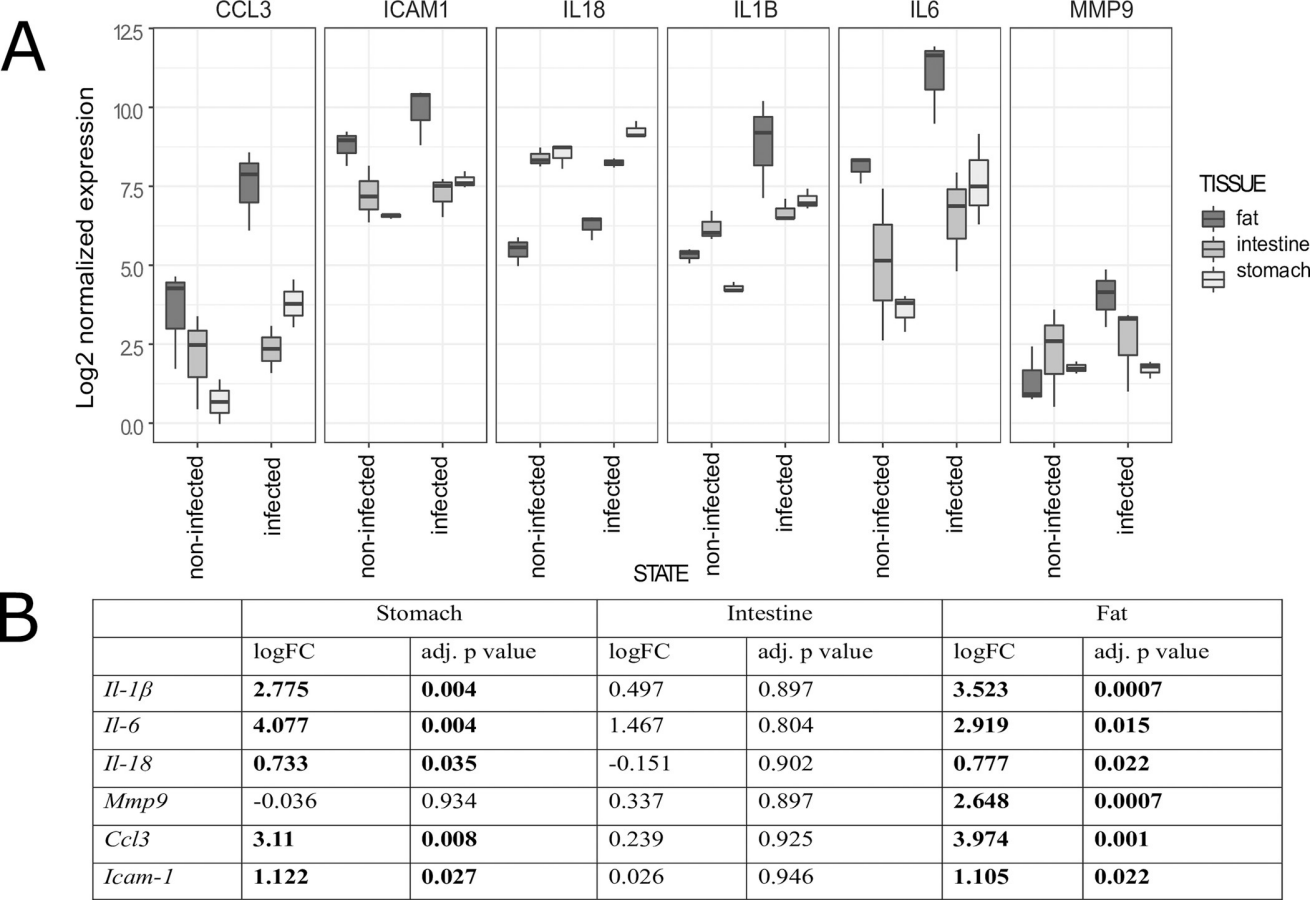
Expression of target genes in tissues of rats experimentally infected with *Anisakis* spp. third-stage larvae. (A) Distribution of target gene expression in stomach, intestine, and visceral adipose tissues grouped according to state (infected or uninfected). Box plots represent distribution of log2-normalized expression; (B) Log2-transformed fold changes of target gene expression with respective adjusted *p*-values.

The expression of target genes was examined in stomach, intestine, and visceral adipose tissue. Out of six tested target genes, five were differentially expressed in stomach, none in intestine, and all six in visceral adipose tissue. In stomach, five genes were upregulated and only *Mmp9* was negligibly downregulated (Fig 6A). The highest fold change was observed for *Il6*, followed by *Ccl3*, *Il1b*, *Icam1*, and *Il18* (Fig 6B). Biologically significant induction was recorded for *Il6*, *Ccl3*, *Il1b*, and *Icam1*. In intestine, four genes were upregulated, with *Il18* being weekly downregulated, and *Icam1* showing no change in expression (Fig 6A). The highest fold change was observed for *Il6*, which was the only biologically significant induction among the target genes (Fig 6B). In visceral adipose tissue, all target genes were upregulated (Fig 6A) with highest fold-change observed for *Ccl3*, followed by *Il1b*, *Il6*, *Mmp9*, *Icam1*, and *Il18* (Fig 6B). Biologically significant induction was recorded for all genes except for *Il18*.

### miRNA expression

**Fig 7:**
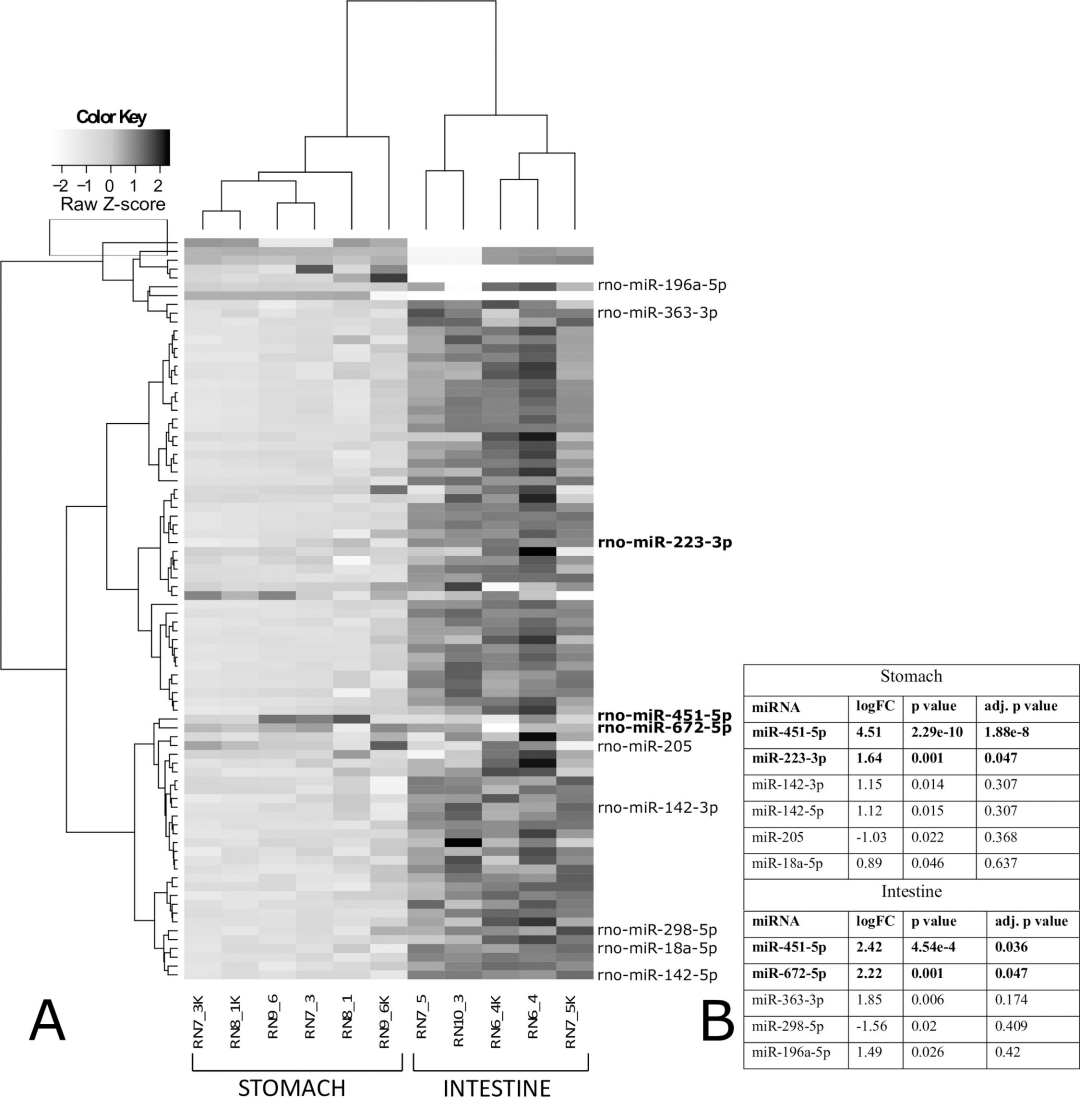
miRNAs expression in stomach and intestine of rats experimentally infected with *Anisakis* spp. third-stage larvae. (A) Two-dimensional hierarchical clustering and heatmap of log2-normalized miRNA expression (Manhattan distance), grouped according to their expression profiles, tissue of origin (stomach and intestine), and tissue state (infected or uninfected). Color key ranges from white to light grey for downregulated (−2 to 0, Z-scores) and from light grey to black for upregulated (0–2, Z-scores) miRNAs. Only differentially expressed miRNAs (*p* < 0.05) in stomach and intestine are listed on the right (adjusted *p*-value < 0.05 in bold). (RN7_3, RN8_1, RN9_6: stomach, infected; RN7_3K, RN8_1K, RN9_6K: stomach, uninfected; RN6_4, RN7_5, RN10_3: intestine, infected; RN6_4K, RN7_5K: intestine, uninfected); (B) Log2-transformed fold changes with respective *p*-values and adjusted *p*-values of differentially expressed miRNAs in stomach and intestine.

miRNA expression was examined in stomach and intestinal tissues (Fig 7A). In stomach, two miRNAs were differentially expressed (*rno-miR-451-5p* and *-223-3p*), both displaying statistical and biological significance (Fig 7A and 7B). Similarly, two miRNAs were differentially expressed in intestine (*rno-miR-451-5p* and *-672-5p*) (Fig 7A and 7B), also displaying statistical and biological significance. When the non-adjusted *p*-value was considered, four additional miRNAs were differentially expressed in stomach; three were upregulated (*rno-miR-142-3p*, *-142-5p*, and *-18a-5p*) and one was downregulated (*rno-miR-205*) (Fig 7B). In intestine, three additional miRNAs were differentially expressed when the non-adjusted *p*-value was considered; two were upregulated (*rno-miR-363-3p* and *-196a-5p*) and one was downregulated (*rno-miR-298-5p*) (Fig 7B). *Rno-miR-451-5p* was the only differentially expressed miRNA in both stomach and intestine.

### DNA methylation

Total methylated DNA was quantified in stomach and intestine. No statistically significant difference (at *p* < 0.05) in DNA methylation was detected between infected (mean 2.56 ng, SD ± 1.03 ng) and uninfected (mean 3.08 ng, SD ± 1.39 ng) stomach tissues or infected (mean 4.25 ng, SD ± 1.80 ng) and uninfected (mean 4.90 ng, SD ± 2.25 ng) intestine tissue of rats, respectively.

## Discussion

Here we present the results of a comprehensive cellular and molecular analysis of the early rat immune response to experimental *Anisakis* spp. infection. A limited number of studies have tackled the pathology in this host-parasite system, concentrating on general histopathological analyses (HE staining, trichrome staining) [35] and more recently tissue transcriptomics [37] and miRNA expression analysis in sera of infected animals [49]. To the best of our knowledge, this is the first comprehensive histopathological analysis of *Anisakis* lesions inferred by classic histopathology, immunofluorescence (IF), and transmission electron microscopy (TEM). In addition, for better insight into the underlying molecular mechanisms of the visible changes, we validated the histology findings by quantification of target genes and miRNA expression in tissues affected by larval migration, and the screening of global DNA methylation levels in *Anisakis*-infected vs. uninfected tissues.

Histopathological analysis of *Anisakis* lesions revealed an acute and generally strong tissue inflammatory response, mostly dominated by neutrophils and macrophages. Both stomach and intestine tissues were hallmarked by extensive submucosal hemorrhages as well as vascular wall and perivascular necrosis, which facilitated leukocyte extravasation through increased vascular permeability. In addition to larva-induced trauma of the tissues accounting for the hemorrhages, *Anisakis* spp. larvae also secrete various proteolytic enzymes [22,50] that degrade surrounding host tissue enhancing larval movement and leading to cellular necrosis. Necrosis is a form of uncontrolled cell death that leads to leakage of various intracellular components, which can further stimulate the inflammatory response [51]. However, the abundance of the observed inflammatory infiltrate differed between stomach and intestine, being markedly elevated in the stomach. The rationale for a such discrepancy could lie in earlier larval penetration through the stomach compared to the intestinal wall, allowing more time for immune cell infiltration of the affected tissues. A similar predominance of neutrophil and macrophage lineages was observed in affected muscle tissue, but with comparatively less inflammatory infiltrate than in stomach. In addition to the later occurrence of larval migration through muscle tissue compared to stomach and consequent later mobilization of immune cells, it appears that the intact blood vessels in the muscle being less permeable to leukocyte extravasation could have added to the infiltrate scarcity. Although helminth infections are usually associated with a marked increase in eosinophil count (eosinophilia) [52], we observed increased numbers of infiltrating eosinophils only in tissues from animals euthanized at later time points (24h, 48 h, and 72h p.i.). Pronounced eosinophil infiltration and subsequent formation of eosinophilic granuloma have been recorded in many cases of human anisakiasis [20,36]. However, eosinophilia is usually not constant at the beginning of clinical manifestations in humans [53] suggesting that the small number of observed eosinophils in rat could be simply due to tissue sampling before the onset of eosinophil infiltration. Nevertheless, eosinophil degranulation occurred early in the course of infection, as revealed by TEM, suggesting that the initial priming has been achieved

Neutrophils are the first cellular component to respond to inflammation and tissue damage, reacting to a wide range of PAMPs or DAMPs, including large and antigenically complex metazoan pathogens. Neutrophil-mediated recruitment of other granulocytes and monocytes at the infection site [54,55] supports our findings. Macrophages have pleiotropic functions, both as immune effector cells and in maintaining tissue homeostasis, including phagocytosis of erythrocytes and clearing of cellular debris [56]. During several helminth infections, a subset of macrophages termed alternatively activated macrophages (AAMs) has been observed [26]. These macrophages are activated in response to T_H_2 cytokines, IL4 and -13, and are characterized by the production of several enzymes such as arginase-1, which is involved in collagen deposition [26,56]. Although their function as effector cells in worm expulsion has been demonstrated in mice infected with the natural gastrointestinal helminth *Heligmosmoides polygyrus* [57], AAM function in wound healing and tissue remodeling caused by large metazoan parasites could be more important than host defense per se [56]. Indeed, in addition to neutrophils, we observed the presence of numerous macrophages. Both cell lineages were associated with necrotic tissue in stomach and muscle. Likewise, TEM revealed the presence of numerous macrophages with engulfed erythrocytes. This high number of macrophages and neutrophils, especially, could also account for the later onset of eosinophil tissue infiltration in the early phase of infection. In the case of *Strongyloides stercolaris*, another intestinal tissue-dwelling nematode, macrophages and neutrophils alone can kill its larvae if soluble factors from either cell type are present [58]. A similar succession of large numbers of neutrophils gradually being replaced by macrophages and eosinophils has also been seen in early *Ascaris* spp. infection in mice [59], although the comparison with *Anisakis* needs to be taken cautiously, as the early infection phase of the former occurs in lungs.

Immunofluorescence of rat sections clearly demonstrated early (6 h p.i.) infiltration of CD3^+^ cells in *Anisakis* lesions. CD3 is a pan T-cell marker, which functions as a TCR co-receptor during activation of different T-cell subsets. A lower number of CD3^+^ cells was seen in both intestine and muscle compared to stomach, which is consistent with the later occurrence of larval migration through these tissues. Interestingly, compared to a single CD3^+^ cell in muscle, the highest number of these cells was seen in the intestine, probably due to the lesion’s proximity to one of the intestinal lymphoid follicles. Helminth infections are typically characterized by strong polarization of T_H_2 immune response [23]. However, in *Trichuris muris* infections, the worm burden can influence the polarization towards either T_H_1 or T_H_2, such that a reduced worm burden promotes the T_H_1 response [60]. Furthermore, in patients with trichinellosis, about half of the peripheral blood mononuclear cells (PBMC) display a T_H_2 profile with the other half displaying either T_H_0 or T_H_1 profiles [61]. In the blood of *Anisakis simplex*-sensitized patients, both T_H_1 and T_H_2 cytokines have been recorded. However, a T_H_1-dominant response is characteristic for patients with gastrointestinal (GI) symptoms [62]. Whether such peripheral immune responses reflect the localized response in the GI tract still remains to be elucidated, requiring detailed immunophenotyping of the infiltrating T-cell populations in *Anisakis* lesions. Immunofluorescent iNOS labeling showed the presence of different iNOS^+^ cells in all three tissues examined. iNOS is expressed by a number of cells such as neutrophils, macrophages, and dendritic cells. High levels of nitric oxide (NO) are produced by macrophages in response to microbial products and T_H_1 cytokines [39], as well as protozoan [63] and metazoan parasites [64,65]. In addition to antimicrobial and antiparasitic effects, NO has cytotoxic effects [39,66], which could partially account for the observed *Anisakis* changes. Interestingly, the strongest iNOS expression was seen in affected muscle in several cell types including neutrophils, fibrocytes, and phagocytes both in epimysium near the site of larval penetration and distantly in the perimysium. High macrophage iNOS production was also recorded in *Trichinella spiralis*-infected mice, adjacent to encysted larva and surrounding muscle fibres, being most pronounced in the late phase of muscle infection [67]. Being able to cross-infect rodents, *Trichinella* spp. naturally reside within their striated muscles, in contrast to *Anisakis* for which rats and humans are naïve accidental hosts. This suggests that, being in an unfavorable environment such as striated muscle, *Anisakis* spp. could induce an early explosive iNOS expression like *Trichinella*, however, with substantial temporal difference. S100A8 and S100A9 are alarmins or DAMPs, which are constitutively expressed in neutrophils, monocytes, and dendritic cells but can also be secreted from activated or damaged macrophages, endothelial cells, fibroblasts, and keratinocytes [40,68]. We observed the presence of S100A8/S100A9^+^ cells in all three tissues examined, with the highest number of cells and the strongest expression in muscle. Unfortunately, with the applied antibody, we were not able to discern between the two proteins expressed, or their complex whatsoever. *In vivo* studies have demonstrated chemotactic activity of S100A8/A9 proteins as early as three hours post-stimulation [69]. The complex influences endothelial integrity through inhibition of several cell-junction proteins [70] thus facilitating leukocyte migration. Given the abundant neutrophil infiltrate and several perivascular necrotic foci in stomach, we unexpectedly identified a negligible number of cells with cytoplasmic expression of these proteins in stomach, mostly localized in the *muscularis externa*. This suggests that the tissue sampling (10 h p.i.) coincided with the cells having already secreted the complex into the extracellular space, affecting its intracellular detection. The detection was further hindered by high autofluorescence of the hemorrhages. Similarly, a relatively small number of S100A8/A9^+^ cells were detected in the intestine, mostly expressed by endothelial cells and possibly occasional fibrocytes. Whether the low number of positive cells is due to early sampling (relative to larval migration time) remains unclear, considering the overall moderate inflammatory infiltrate. In muscle tissue, we found various S100A8/A9^+^ cells, most likely fibrocytes, neutrophils, and occasional eosinophils, which is consistent with previous reports [68,71]. However, the very strong expression in myocytes is puzzling. While expression of these proteins can be induced in murine vascular smooth muscle cells or cardiomyocytes following inflammatory stimuli such as bacterial infection [72], we failed to find references demonstrating expression of these markers at the protein level in skeletal muscle. However, in a recently published transcriptome analysis of *A*. *pegreffii*-infected rats, S100A8 and S100A9 were among the most highly expressed genes [37]. S100A8/A9 protein expression during helminth infection (particularly in neutrophils), has been demonstrated in lung inflammation in mice following infection with *Litomosoides sigmodontis* L3 larvae [73] as well as at the mRNA level in sheep infected with the digenean *Fasciola hepatica* [74].

Acute inflammation, such as in a rat model of *Anisakis* spp. infection, is characterized by production of numerous cytokines and signaling molecules that govern the immune response and wound healing after tissue damage. Here, we assessed the expression of several common inflammatory markers (i.e., *Il1b*, *Il6*, *Il18*, *Ccl3*, and *Icam1*, as well as *Mmp9*, which is involved in tissue remodeling [75]). Interestingly, while all markers except *Mmp9* were differentially expressed in adipose and stomach tissues, respectively, no difference was detected in the intestine. As mentioned earlier, severe submucosal hemorrhages were present both in stomach and intestine following larval migration. Hemorrhages have been shown to potentiate the expression and secretion of proinflammatory cytokines, including IL-1 beta and IL-6, as well as delay wound healing following trauma [76]. In contrast, the lack of significant induction of these genes in the intestine is likely due to the early sampling in respect to larval penetration, corresponding to observed mild to moderate inflammatory infiltrate in paraffin sections. However, the possibility that proinflammatory cytokines other than those evaluated are induced earlier in response to *Anisakis* migrating larvae should not be excluded. Surprisingly, all six genes were differentially expressed in visceral adipose tissue, with all but *Il18* displaying a biologically significant induction. In addition to the most abundant adipocytes, adipose accumulations encompass various tissue types, endothelial cells, smooth muscle cells, and fibroblast as well as almost all immune cells including resident macrophages, neutrophils, T cells, and B cells, to name a few [77]. In addition to energy storage, adipose tissues have an important role in regulating both local and systemic inflammation through secretion of anti- and proinflammatory cytokines, including IL-1 beta, IL-6, and IL-18 [78], as well as MMP9 [79]. However, at this point, it remains unclear whether such strong induction of proinflammatory genes in adipose tissue is due to the mere presence of *Anisakis* larva, tissue damage and inflammation in adjoining tissue or both. In stomach, *Mmp9* was the only gene that showed virtually no difference in expression compared to control. MMP9 is known to be involved in tissue remodeling and wound repair; however, wound healing might be delayed by hemorrhage through increased production of proinflammatory cytokines [76]. The lack of change in *Mmp9* expression could be the result of delayed induction due to severe submucosal hemorrhages. In addition, MMP9 is suggested to be important for neutrophil migration through basement membrane [80], congruent with our histology data, as we found no intraepithelial infiltration of neutrophils, likely due to no *Mmp9* expression at this site. All three proinflammatory cytokines (i.e. *Il1b*, *Il6*, and *Il18*) were differentially expressed in stomach, although *Il18* did not display biologically significant induction. Both IL-1β and IL-18 are activated and secreted from multiprotein complexes called inflammasomes, containing one of the NOD-like receptors (NLRs), such as NLRP3 [81]. The difference in expression of these two genes could, therefore, be due to factors other than the mechanism of activation shown in the mouse model of *Trichuris muris* infection, where both the worm and its secreted products induced NLRP3-dependent production of IL-18 [82]. IL-18 induces T_H_1 polarization through secretion of interferon-gamma (IFN-γ), except in the absence of IL-12 or IL15, when it promotes T_H_2 polarization [83]. Indeed, *T*. *spiralis*-infected IL-18 knockout mice had significantly higher production of T_H_2 cytokines with lower worm burden, compared to wild type-mice [84]. Nevertheless, induction of *Il18* and its role in driving the T_H_1 response could indicate that early rat responses to *Anisakis* spp. are of mixed T_H_1/T_H_2 type possibly with T_H_2 predominance, similar to the intestinal phase of mouse *T*. *spiralis* infection [85]. IL-1β is a major endogenous pyrogen, which activates neutrophils and macrophages for phagocytosis of invading pathogens and production of oxygen and nitrogen radicals. In addition, it stimulates production of other proinflammatory cytokines, such as tumor necrosis factor α (TNFα) and IL-6 [81]. However, in the context of helminth resistance, IL-1 beta can have contrasting effects. While it attenuates the protective T_H_2 response and promotes parasite chronicity in *H*. *polygyrus* infection [86], it induces the T_H_2 response and parasite expulsion in *T*. *muris* infection [87]. How IL-1 beta affects immunity during *Anisakis* infection remains unclear because the parasite fails to achieve adult stage in the accidental host, which would warrant chronic inflammation, or has a low chance of re-infecting the same accidental host over a longer time span. Remarkably strong expression was observed for *Il6* (logFC = 4.07). IL-6 is another major endogenous pyrogen, which plays a pivotal role in the immune response by switching from neutrophil to monocyte recruitment, skewing monocyte differentiation into macrophages and T-cell expression and differentiation to T_H_2 and T_H_17 phenotypes [88]. However, mice infected with *H*. *polygyrus* had a suppressed T_H_2 response and higher number of FoxP3^+^ cells compared to IL-6-deficient mice [89], indicating a pleiotropic role for this cytokine in regulating the immune system. Interestingly, we noted a very strong expression of *Ccl3*, which exceeded the expression of *Il1b*. CCL3 is a chemokine with pyrogenic properties, but more importantly is a chemoattractant for neutrophils and an activator of their oxidative burst [90]. In contrast to S100A8/A9^+^ cells, which were observed in small numbers, the strong *Ccl3* expression likely accounted for the abundant neutrophil infiltration observed in *Anisakis* lesions. Although significant, *Icam1* displayed moderate expression compared to the aforementioned markers. ICAM1 is expressed by endothelial cells and upon inflammatory stimulus leads to increased leukocyte transmigration and increased vascular permeability through downregulation of cell-junction proteins [91]. ICAM1 regulates adhesion of neutrophils to endothelium and their transendothelial migration via either paracellular or transcellular routes [92]. Therefore, the abundant neutrophil infiltration is likely a result of the combined effects of CCL3 and ICAM1, although the latter showed four-fold lower expression than the former. This discrepancy in expression is possibly due to vascular wall necrosis leading to a partial loss of *Icam1* mRNA. Both live L3 *Anisakis* larvae and protein crude extract (CE), which simulates dying larva, were recently found to stimulate human monocyte-derived dendritic cells (DCs) to secrete CCL3, IL-6, soluble ICAM1 (sICAM1), and IL-1 alpha, another isoform of IL-1 with properties overlapping IL-1 beta [93], thus, confirming our findings.

MicroRNA expression analysis was performed in stomach and intestine tissues affected by *Anisakis* larval migration. In total, three miRNAs were differentially expressed, two in stomach (*rno-miRNA-451-5p* and *-223-3p*) and two in intestine (*rno-miRNA-451-5p* and *-672-5p*), with *rno-miRNA-451-5p* being expressed in both tissues. To the best of our knowledge, this is the first miRNA expression analysis performed in tissue samples directly affected by larval migration, rather than in sera of infected animals as reported by Corcuera *et al*. (2018). The authors found two differentially expressed miRNAs in rat sera, in addition to four others based on RQ values, which were different to those we identified in tissues. However, this could be attributed to the mode of infection (live L3 vs. crude extract inoculated into gastric mucosa), duration of infection (6 h p.i. vs. 2.5 months) and sample type (affected tissues vs. serum). In *Anisakis* stomach lesions, *miRNA-451* displayed particularly strong expression (logFC = 4.51, adj. *p* = 1.88e^-8^) and roughly four-fold lower expression in intestine (logFC = 2.42, adj. *p* = 0.036). miRNA-451 has been shown to downregulate CD4^+^ T-cell proliferation, as evidenced by mice infected with *Plasmodium yoelli* having significantly lower numbers of CD4^+^ cells in addition to higher parasite burden compared to *miR-451*^*-/-*^ mice [94]. Furthermore, miR-451 impairs neutrophil chemotaxis [95] and negatively affects secretion of IL-6 and CCL3 [96]. In stomach, we observed abundant neutrophil infiltration as well as strong expression of both *Il6* and *Ccl3*. IL-6 has been shown to positively regulate expression of *miR-451*, potentially forming a regulatory loop that culminates in decreased IL-6 expression [96]. Therefore, the expression of *miR-451* in stomach is likely the consequence of strong positive feedback of *Il6* expression, aimed at the downregulation of over expressed *Il6*. Interestingly, mild inflammatory infiltrate coupled with the lack of statistically significant expression of the target genes was seen in intestine, although *miR-451* was significantly expressed. Nevertheless, we observed biologically significant induction of *Il6* (logFC = 1.47), which might have been sufficient to drive initial expression of this particular miRNA. Although being expressed eight-fold lower than *miR-451*, *miR-223* showed significantly higher expression in stomach compared to control. It was reported that absence of miR-223 drives accelerated recruitment of myeloid cells (i.e. polymorphonuclear leukocytes and mononuclear phagocytes) at the site of infection, and subsequent tissue damage [97]. Therefore, we hypothesize that induction of this miRNA serves to limit neutrophil infiltration and consequent tissue damage, and gradual substitution with other immune cells. Furthermore, miRNA-223 has also been reported to directly target *Ccl3* and *Il6* [97]. In addition, miRNA-223 has been reported to negatively regulate the production of IL-1 beta by repression of the NLRP3 inflammasome [98]. Taken together, it appears that *miRNA-223* could play an important role in shaping *Anisakis* pathology despite substantially lower expression compared to miR-451. However, IL-6 itself is able to downregulate expression of miRNA-223 [99]. Therefore, the difference in expression between *miRNA-223* and *miR-451* could be attributed to strong *Il6* expression. Intestinal expression of *miR-672* during *Anisakis* infection remains enigmatic, as does its role in regulation of mRNA in general. Namely, data on the function of miR-672 is scarce, except for evidence of its modulation in a murine model of acute and chronic asthma [100]. Considering the substantial difference in inflammatory infiltrate between stomach and intestine and modest downregulation of this miRNA in stomach, we can only assume that the quantity and type of immune cells infiltrating the two organs account for *miR-672* induction in the intestine.

In addition to post-transcriptional regulation via miRNAs, gene expression can be regulated by chemical modifications, such as DNA methylation, which prevents binding of transcription factors by introducing changes in promoter regions [34]. Moreover, aberrant DNA methylation has been associated with several types of cancer due to repression of tumor-suppressor genes [101]. In this study, we screened for changes in global DNA methylation status and found no significant differences between infected and uninfected tissue. We argue that despite our results, DNA methylation could indeed have a role in the immune response to *Anisakis* infection, but the effect is probably reserved for prolonged *Anisakis* infection or to specific genes non-discernible by performed design. In a murine model of urogenital schistosomiasis, changes in DNA methylation levels coincided with urothelial hyperplasia, a key preneoplastic lesion leading to schistosomal bladder cancer [102]. Unlike *S*. *haematobium*, *Anisakis* spp. are not considered carcinogenic, but their potential role in carcinogenesis has been controversial ever since it was suggested that *A*. *simplex* might be a co-factor for the development of gastric cancer [103]. Interestingly, Sonoda *et al*. [104] summarized 27 cases reporting attachment of *Anisakis* larva to GI cancer, and given the role of methylation in carcinogenesis, this interaction warrants further research.

Taken together, our results show that *Anisakis* spp. trigger a rapid and robust immune response in rats, characterized by abundant neutrophil and macrophage infiltration and significant induction of *Il6* and *Il1b* proinflammatory cytokines and the chemokine *Ccl3*, which were among the most highly expressed genes of more than 1300 differentially expressed genes in the transcriptome of *Anisakis pegreffii*-infected rats [37]. Moreover, three miRNAs were differentially expressed, indicating their involvement in particular mRNA expression profiles measured in affected tissues. However, the functional role of these miRNAs in *Anisakis* infection remains unknown. In contrast, no changes in DNA methylation status were observed between infected and uninfected tissue tissues. Establishing chronic infection and employing different methodological approaches to study the functional role of miRNAs and DNA methylation will help to address some of the controversies surrounding *Anisakis* infection in humans.

## Supporting information

S1 Table List of samples used for each downstream analysis. (PHD-classic histopathology; IF-immunofluorescence; TEM-transmission electron microscopy; miRNA-miRNA profiling; TGE-target gene expression; DNAm-DNA methylation)
(DOCX)

S1 Fig Representative micrographs showing uninfected tissue controls. (A) Stomach, cardiac region; (B) Stomach, body, greater curvature; (C) Intestine; (D) Muscle. HE, scale bar = 200 μm.
(TIF)

S2 Fig Representative micrographs of CD3 immunolabeling of uninfected control tissue. (A, B, C) Stomach; (C, D, E) Intestine; (E, F, G) Muscle. Immunofluorescence, scale bar: (A, B, C, G, H, I) = 20 μm, (D, E, F) = 10 μm.
(TIF)

S3 Fig Representative micrographs of negative stain control for CD3 antibody. (A, B, C) Stomach; (D, E, F) Intestine; (G, H, I) Muscle. Immunofluorescence, scale bar = 10 μm.
(TIF)

S4 Fig Representative micrographs of iNOS immunolabeling of uninfected control tissue. (A, B, C) Stomach; (C, D, E) Intestine; (E, F, G) Muscle. Immunofluorescence, scale bar = 10 μm.
(TIF)

S5 Fig Representative micrographs of negative stain control for iNOS antibody. (A, B, C) Stomach; (D, E, F) Intestine; (G, H, I) Muscle. Immunofluorescence, scale bar = 10 μm.
(TIF)

S6 Fig Representative micrographs of S100A8/A9 (MRP8+MRP14) immunolabeling of uninfected control tissue. (A, B, C) Stomach; (C, D, E) Intestine; (E, F, G) Muscle. Immunofluorescence, scale bar = 10 μm.
(TIF)

S7 Fig Representative micrographs of negative stain control for S100A8/A9 (MRP8+MRP9) antibody. (A, B, C) Stomach; (D, E, F) Intestine; (G, H, I) Muscle. Immunofluorescence, scale bar = 10 μm.
(TIF)
