# Supplementary material for: Interplay between proinflammatory cytokines, miRNA, and tissue lesions in Anisakis-infected Sprague-Dawley rats
Source: PLoS Negl Trop Dis. 2019 May 15;13(5):e0007397. doi: 10.1371/journal.pntd.0007397 (PMC6538193; doi:10.1371/journal.pntd.0007397)
Supplement: S1 Table — (PHD-classic histopathology; IF-immunofluorescence; TEM-transmission electron microscopy; miRNA-miRNA profiling; TGE-target gene expression; DNAm-DNA methylation) (DOCX) [file pntd.0007397.s001.docx]

| **Preliminary experiment** | |  |  |  |
| --- | --- | --- | --- | --- |
| **Sample ID** | **Description** | | **Sampling hour** | **Analysis** |
| RN1 | Stomach/infected | | 6 | PHD/IF |
| RN2 | Stomach/infected | | 6 | PHD/IF |
| RN3.2 | Stomach/infected | | 6 | PHD/IF |
| RN5.1 | Muscle/infected | | 10 | PHD/IF/TEM |
| RN5.2. | Stomach/infected | | 10 | PHD/IF |
| RN6.1 | Stomach/infected | | 10 | PHD/IF |
| RN6.2 | Stomach/infected | | 10 | PHD/IF |
| RN6.4 | Intestine/infected | | 10 | PHD/IF/TEM |
| RN9.1 | Coecum/infected | | 24 | PHD/IF/TEM |
| RN9.2 | Stomach/infected | | 24 | PHD/IF/TEM |
| RN10.1 | Stomach/infected | | 48 | PHD/IF |
| RN10.2 | Stomach/infected | | 48 | PHD/IF |
| RN10.3 | Stomach/infected | | 48 | PHD/IF |
| RN12 | Stomach/infected | | 48 | PHD/IF |
| RN13.1 | Stomach/infected | | 72 | PHD/IF |
| RN13.2 | Stomach/infected | | 72 | PHD/IF |
| RN15 | Stomach/infected | | 72 | PHD/IF |
| **Main experiment** | |  |  |  |
| RN6-2 | Stomach/infected | | 6 | DNAm |
| RN7-3 | Stomach/infected | | 6 | miRNA/TGE/DNAm |
| RN8-1 | Stomach/infected | | 6 | miRNA/TGE/DNAm |
| RN9-6 | Stomach/infected | | 6 | miRNA/TGE/DNAm |
| RN9-7 | Stomach/infected | | 6 | DNAm |
| RN6-2K | Stomach/non-infected | | 6 | DNAm |
| RN7-3K | Stomach/non-infected | | 6 | miRNA/TGE/DNAm |
| RN8-1K | Stomach/non-infected | | 6 | miRNA/TGE/DNAm |
| RN9-6K | Stomach/non-infected | | 6 | miRNA/TGE/DNAm |
| RN9-7K | Stomach/non-infected | | 6 | DNAm |
| RN6-4 | Intestine/infected | | 6 | miRNA/TGE/DNAm |
| RN7-5 | Intestine/infected | | 6 | miRNA/TGE/DNAm |
| RN8-3 | Intestine/infected | | 6 | DNAm |
| RN10-3 | Intestine/infected | | 6 | miRNA/TGE/DNAm |
| RN6-4 | Intestine/non-infected | | 6 | miRNA/TGE/DNAm |
| RN7-5 | Intestine/non-infected | | 6 | miRNA/TGE/DNAm |
| RN8-3K | Intestine/non-infected | | 6 | DNAm |
| RN10-3 | Intestine/non-infected | | 6 | miRNA/TGE/DNAm |
