# Supplementary figures and images for: Interplay between proinflammatory cytokines, miRNA, and tissue lesions in Anisakis-infected Sprague-Dawley rats
Source: PLoS Negl Trop Dis. 2019 May 15;13(5):e0007397. doi: 10.1371/journal.pntd.0007397 (PMC6538193; doi:10.1371/journal.pntd.0007397)

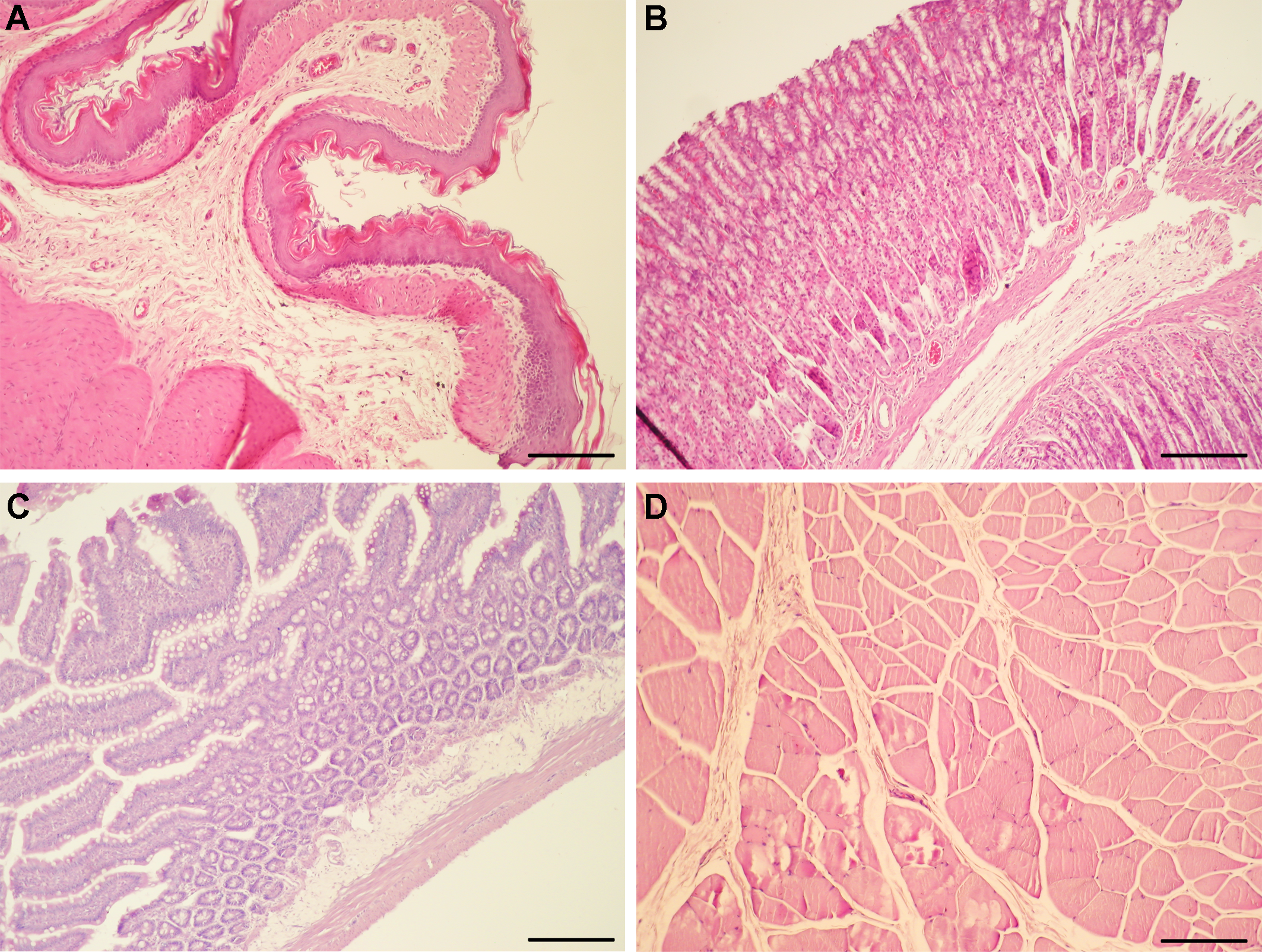

Supplement: S1 Fig — (A) Stomach, cardiac region; (B) Stomach, body, greater curvature; (C) Intestine; (D) Muscle. HE, scale bar = 200 μm. (TIF) [file pntd.0007397.s002.tif]

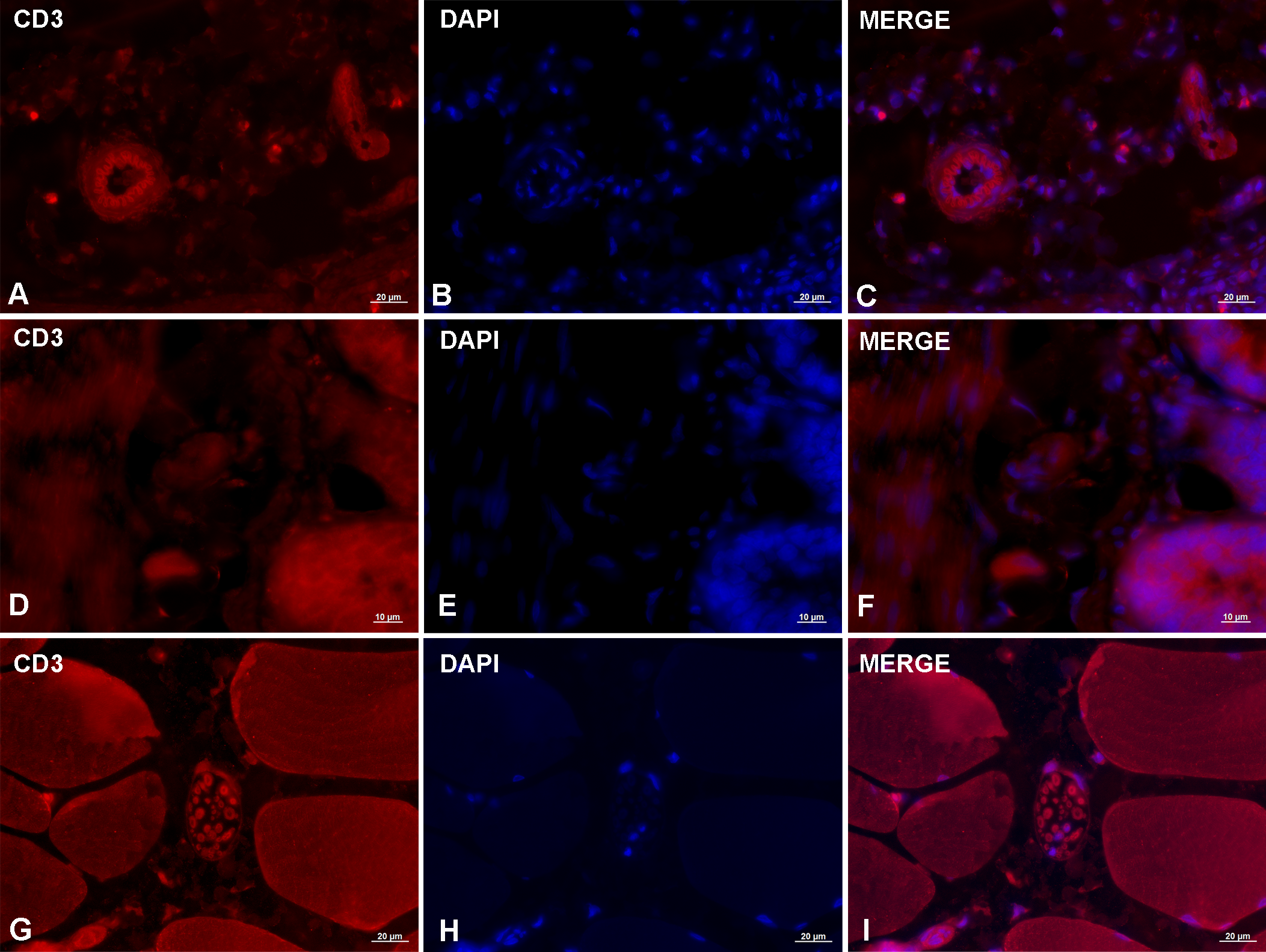

Supplement: S2 Fig — (A, B, C) Stomach; (C, D, E) Intestine; (E, F, G) Muscle. Immunofluorescence, scale bar: (A, B, C, G, H, I) = 20 μm, (D, E, F) = 10 μm. (TIF) [file pntd.0007397.s003.tif]

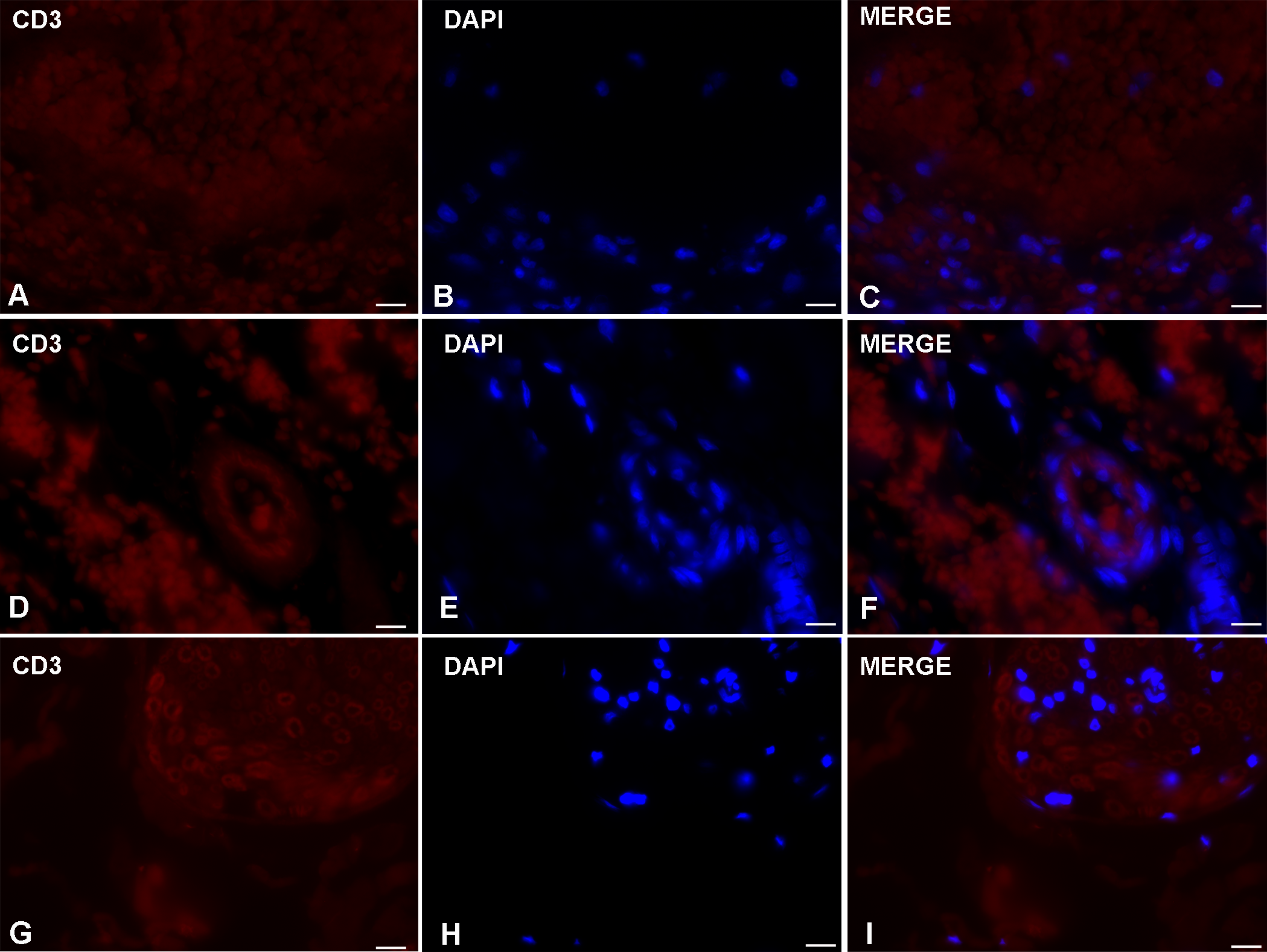

Supplement: S3 Fig — (A, B, C) Stomach; (D, E, F) Intestine; (G, H, I) Muscle. Immunofluorescence, scale bar = 10 μm. (TIF) [file pntd.0007397.s004.tif]

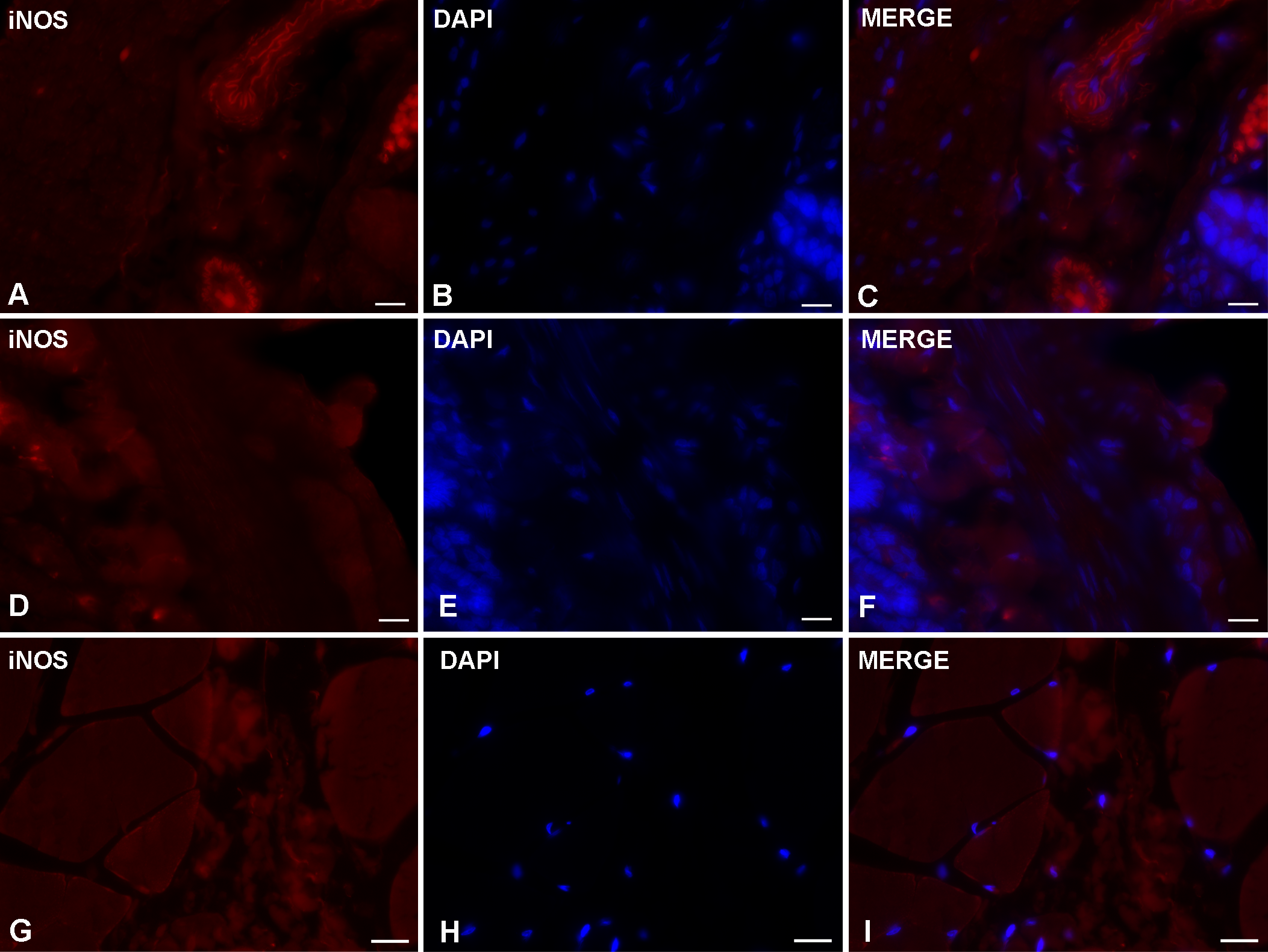

Supplement: S4 Fig — (A, B, C) Stomach; (C, D, E) Intestine; (E, F, G) Muscle. Immunofluorescence, scale bar = 10 μm. (TIF) [file pntd.0007397.s005.TIF]

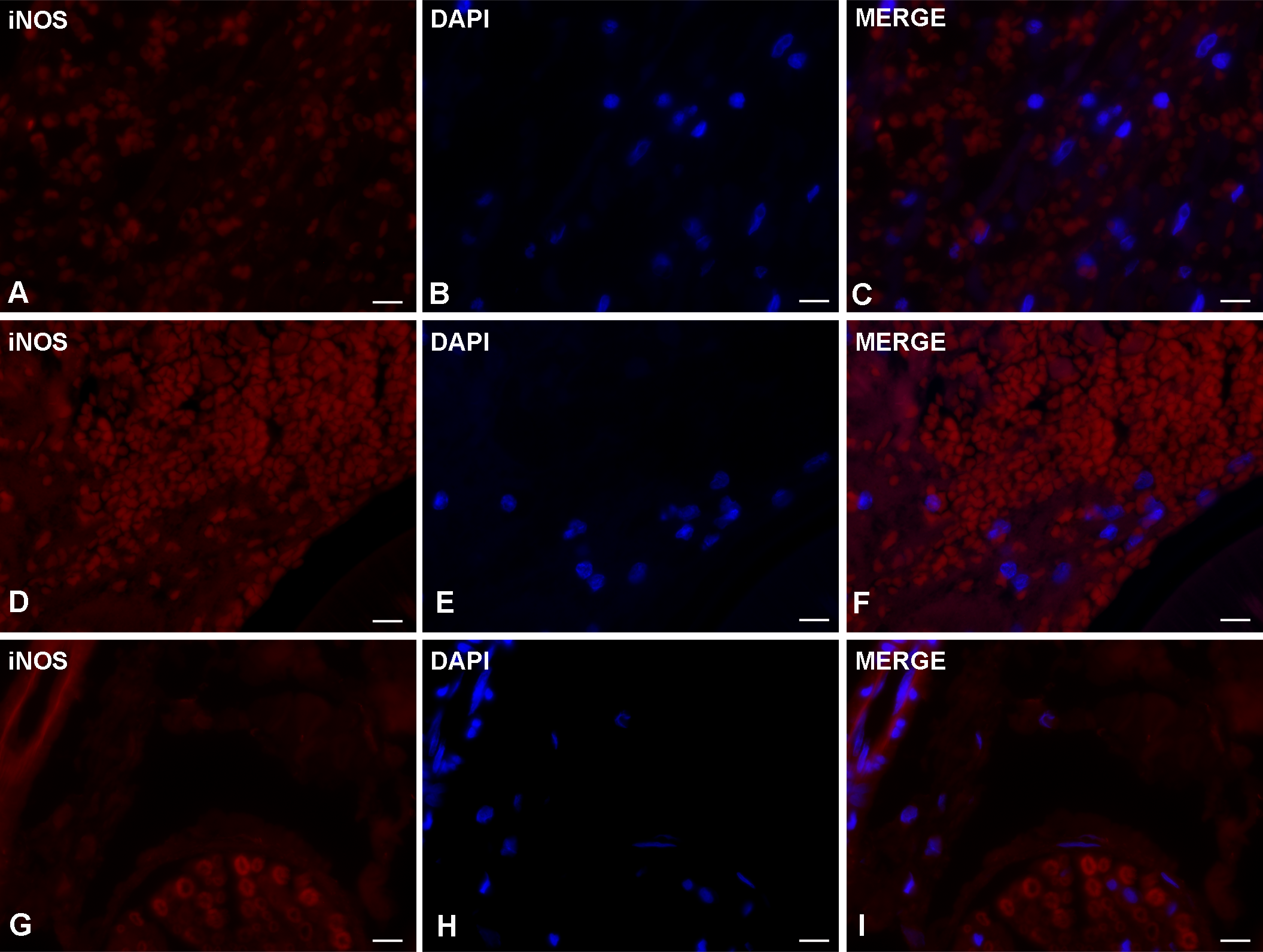

Supplement: S5 Fig — (A, B, C) Stomach; (D, E, F) Intestine; (G, H, I) Muscle. Immunofluorescence, scale bar = 10 μm. (TIF) [file pntd.0007397.s006.tif]

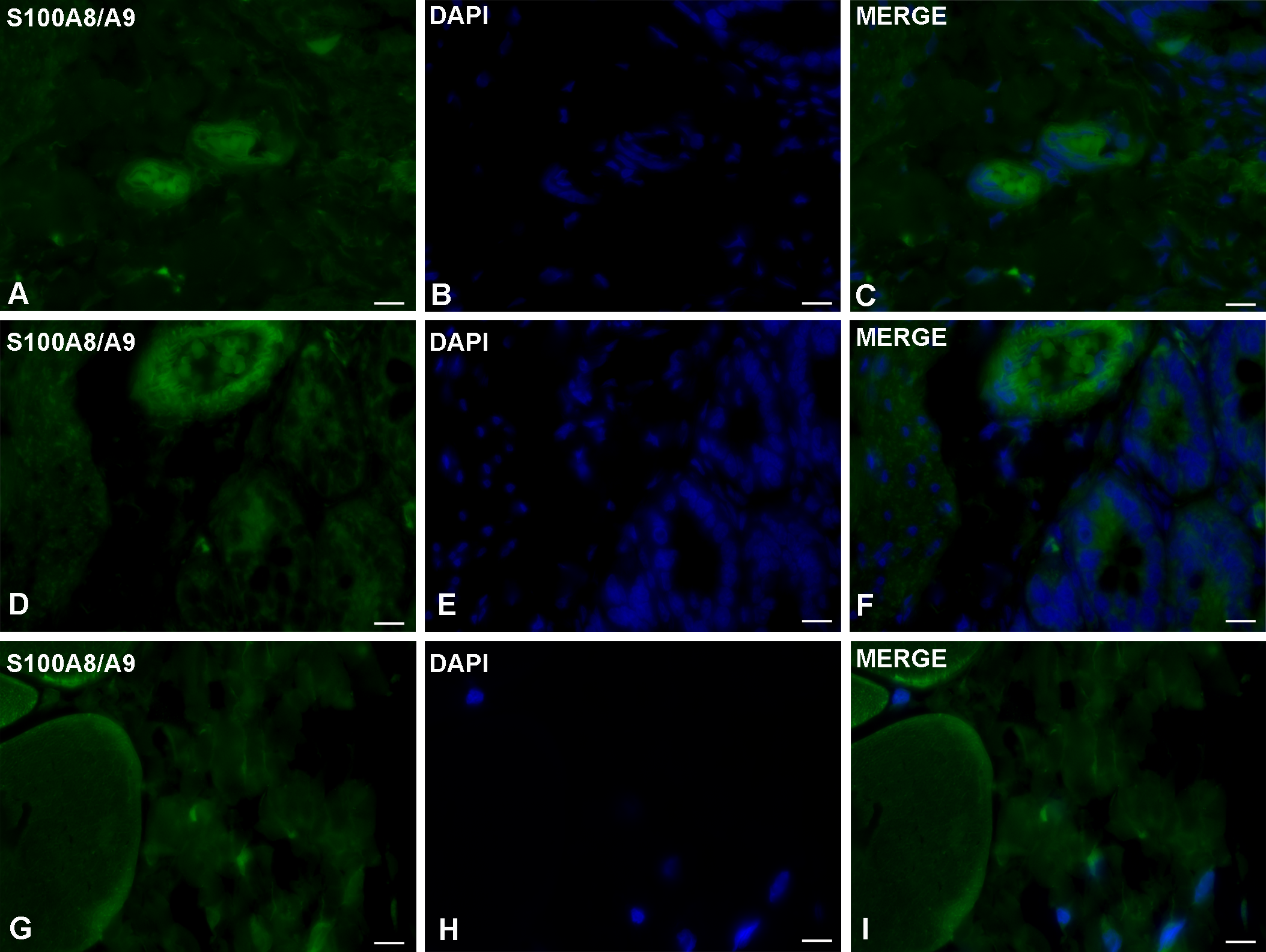

Supplement: S6 Fig — (A, B, C) Stomach; (C, D, E) Intestine; (E, F, G) Muscle. Immunofluorescence, scale bar = 10 μm. (TIF) [file pntd.0007397.s007.tif]

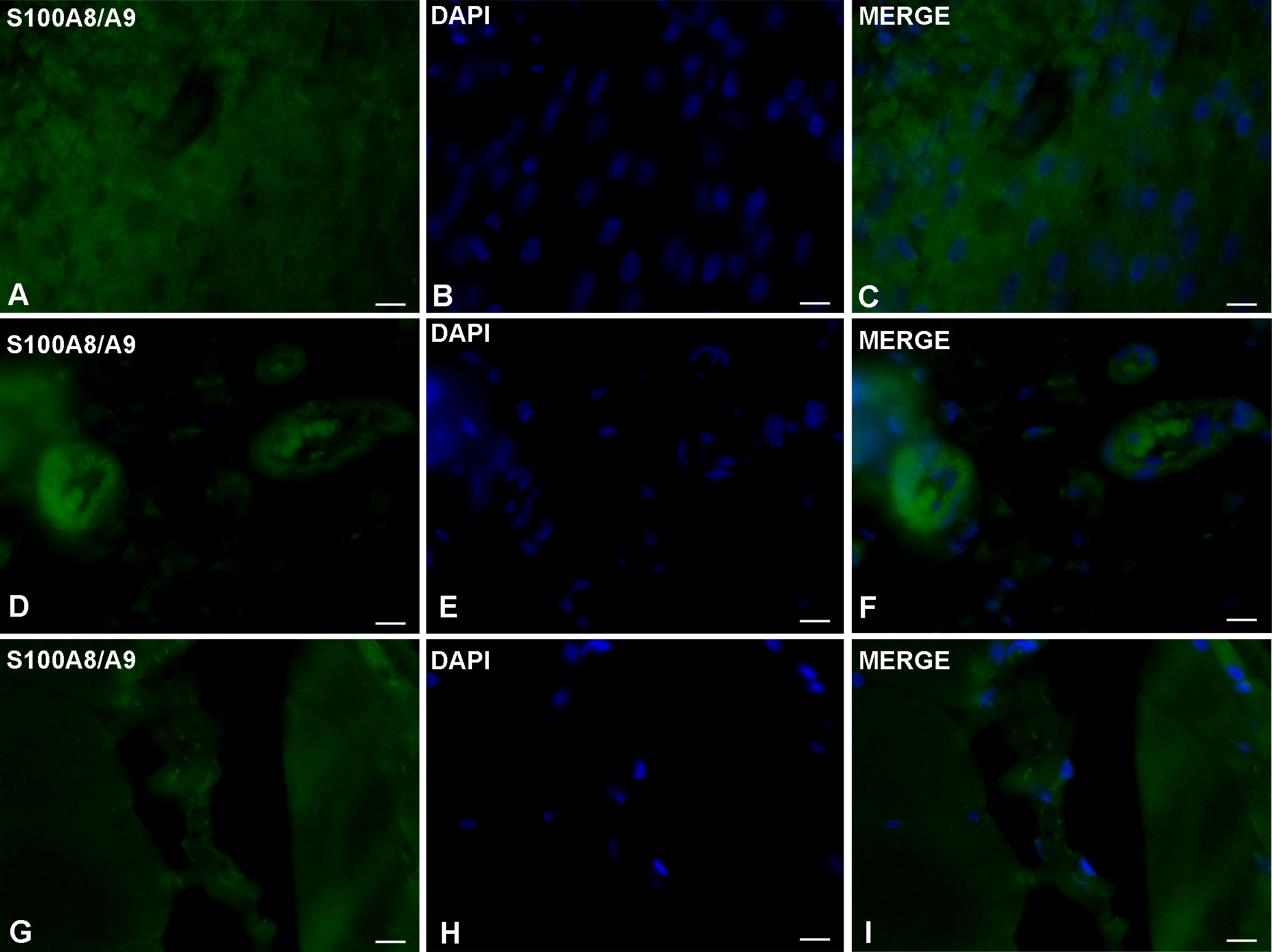

Supplement: S7 Fig — (A, B, C) Stomach; (D, E, F) Intestine; (G, H, I) Muscle. Immunofluorescence, scale bar = 10 μm. (TIF) [file pntd.0007397.s008.tif]
